# Inflammatory crosstalk impairs phagocytic receptors and aggravates atherosclerosis in clonal hematopoiesis in mice

**DOI:** 10.1172/JCI182939

**Published:** 2024-11-12

**Authors:** Wenli Liu, Brian D. Hardaway, Eunyoung Kim, Jessica Pauli, Justus Leonard Wettich, Mustafa Yalcinkaya, Cheng-Chieh Hsu, Tong Xiao, Muredach P. Reilly, Ira Tabas, Lars Maegdefessel, Kai Schlepckow, Christian Haass, Nan Wang, Alan R. Tall

**Affiliations:** 1 Division of Molecular Medicine, Department of Medicine, and; 2 Division of Cardiology, Department of Medicine, Columbia University, New York, New York, USA.; 3 Institute of Molecular Vascular Medicine, Klinikum rechts der Isar, Technical University of Munich, Munich, Germany.; 4 German Center for Cardiovascular Research (DZHK), partner site Munich Heart Alliance, Germany.; 5 Department of Medicine, Karolinska Institute, Stockholm, Sweden.; 6 German Center for Neurodegenerative Diseases (DZNE), Munich, Germany.; 7 Metabolic Biochemistry, Biomedical Center, Faculty of Medicine, Ludwig-Maximilians University, Munich, Germany.; 8 Munich Cluster for Systems Neurology (SyNergy), Munich, Germany.

**Keywords:** Vascular biology, Atherosclerosis

## Abstract

Clonal hematopoiesis (CH) increases inflammasome-linked atherosclerosis, but the mechanisms by which CH mutant cells transmit inflammatory signals to nonmutant cells are largely unknown. To address this question, we transplanted 1.5% *Jak2^V617F^* (*Jak2^VF^*) bone marrow (BM) cells with 98.5% WT BM cells into hyperlipidemic *Ldlr^–/–^* mice. Low-allele-burden (LAB) mice showed accelerated atherosclerosis with increased features of plaque instability, decreased levels of the macrophage phagocytic receptors c-Mer tyrosine kinase (MERTK) and triggering receptor expressed on myeloid cells 2 (TREM2), and increased neutrophil extracellular traps (NETs). These changes were reversed when *Jak2^VF^* BM was transplanted with *Il1r1^–/–^* BM. LAB mice with noncleavable MERTK in WT BM showed improvements in necrotic core and fibrous cap formation and reduced NETs. An agonistic TREM2 antibody (4D9) markedly increased fibrous caps in both control and LAB mice, eliminating the difference between the groups. Mechanistically, 4D9 increased TREM2^+^PDGFB^+^ macrophages and PDGF receptor-α^+^ fibroblast–like cells in the cap region. *TREM2* and *PDGFB* mRNA levels were positively correlated in human carotid plaques and coexpressed in macrophages. In summary, low frequencies of *Jak2^VF^* mutations promoted atherosclerosis via IL-1 signaling from *Jak2^VF^* to WT macrophages and neutrophils, promoting cleavage of phagocytic receptors and features of plaque instability. Therapeutic approaches that stabilize MERTK or TREM2 could promote plaque stabilization, especially in CH- and inflammasome-driven atherosclerosis.

## Abstract 2

Low frequency *Jak2^VF^* mutations promote atherosclerosis via IL-1 mediated cross-talk. Therapeutic approaches that increase MERTK or TREM2 could promote plaque stabilization in inflammasome-driven atherosclerosis.

## Graphical abstract

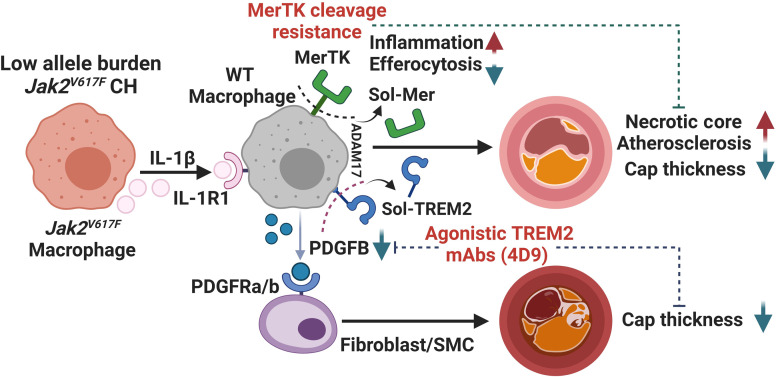

## Introduction

Although LDL-lowering treatments have been proven effective, atherosclerotic cardiovascular disease (CVD) remains the leading cause of morbidity and mortality in the United States, accounting for 28% of all deaths (1). Despite achieving considerable reductions in LDL cholesterol in clinical studies (2–4), a significant residual cardiovascular risk remains, highlighting the need for potential treatment alternatives. Recent clinical trials utilizing antiinflammatory therapies, especially IL-1β antibodies (5) or colchicine (6), have demonstrated a decrease in CVD events. However, these treatments were associated with an increased risk of infections. IL-1β antibodies remain unapproved for CVD indications, and although colchicine was recently approved by the FDA, its therapeutic potential may be limited (7). This indicates an urgent need for additional antiinflammatory treatments targeted for patients with high levels of inflammatory risk, identified either by biomarkers or by genetic factors.

Clonal hematopoiesis (CH) has recently been identified as an important genetic risk factor for CVD (8). This condition results from somatic mutations in leukemogenic genes, providing a survival advantage to hematopoietic stem cells and causing the clonal expansion of blood cells (9, 10). While CH is associated with an increased risk of hematological malignancy, it is much more commonly associated with coronary heart disease, premature myocardial infarction, thrombotic stroke, and heart failure (8, 11, 12). CH of indeterminate potential (CHIP) is identified by the existence of a leukemogenic gene mutation exhibiting a variant allele frequency (VAF) of no less than 2% in peripheral blood, without clinical indications of hematological malignancy (13). Targeted and deeper DNA sequencing methods reveal higher frequencies of CH mutations (14). CVD risk associated with *TET2* and *DNMT3A* CHIP increases with VAF and is only significantly increased at a VAF of greater than 10% (15); however, detection sensitivity and accuracy may have been limited (16). Targeted sequencing has shown progression of heart failure in CH at a VAF of less than 5% (17, 18). An increase in incident coronary heart disease events in CH at a VAF of 0.5%–2% has recently been reported in East Asian cohorts (19).

*JAK2^V617F^* (*JAK2^VF^*) increases hematopoietic cytokine signaling and is a frequent driver mutation in myeloproliferative neoplasms (MPNs) (20). *JAK2^VF^* CH, while less common than *DNMT3A*, *TET2*, or *ASXL1* CH, shows the strongest association with CVD. In a high-risk population, the increased CVD risk for *JAK2^VF^* was 12-fold (8). In a recent meta-analysis of relatively healthy individuals in the United Kingdom Biobank, Mass General Brigham Biobank, and all of the US Biobank, CH overall significantly increased CVD risk by approximately 1.1-fold, whereas the increased risk for *JAK2^VF^* was 2-fold. The increased CVD risk was also found in individuals without overt MPN or altered blood cell counts (13, 21). While formerly considered rare, *Jak2^VF^* has been detected in 3.1% of a general European population using digital droplet PCR (22) and associates with thrombotic risk at low allele burden (LAB) (2%) (22, 23). In the European population, the *JAK2^VF^* mutation was found in 11% of patients with ischemic stroke versus 4% in matched controls (24). *JAK2^VF^* is present at similar frequencies in men and women and in different ethnic groups in the US population (24, 25). Thus, *JAK2^VF^* is emerging as an important CVD risk factor. However, the effect of low VAF *JAK2^VF^* on atherosclerosis remains poorly understood.

Although macrophages play a key role in the progression of atherosclerosis, distinct macrophage populations that express phagocytic receptors may have antiinflammatory, pro-resolving properties (26). For example, c-Mer tyrosine kinase (MERTK), an efferocytosis receptor, reduces necrotic core formation and increases fibrous caps in advanced plaques (27). griggering receptor expressed on myeloid cells 2 (TREM2) is a lipid/lipoprotein-activated phagocytic molecule that promotes microglial or macrophage viability and expression of genes involved in efferocytosis, lysosomal catabolism, and cholesterol efflux (28, 29). Trem2^hi^ macrophages take up modified LDL, become foam cells with low expression of inflammatory genes, and have a potential beneficial role in atherogenesis (30–33). The goals of the present study were to assess atherogenesis in a hyperlipidemic, LAB model of *Jak2^VF^*, then to define the mechanisms of accelerated atherosclerosis, especially those involving IL-1β–mediated crosstalk from mutant to WT myeloid cells that led to reduced levels of MERTK and TREM2. We showed that interventions that stabilized these receptors produced improvements in features of plaque stability, establishing causation and suggesting alternative therapeutic interventions.

## Results

### LAB Jak2^VF^ CH increases atherosclerosis and features of plaque instability.

**Figure 1:**
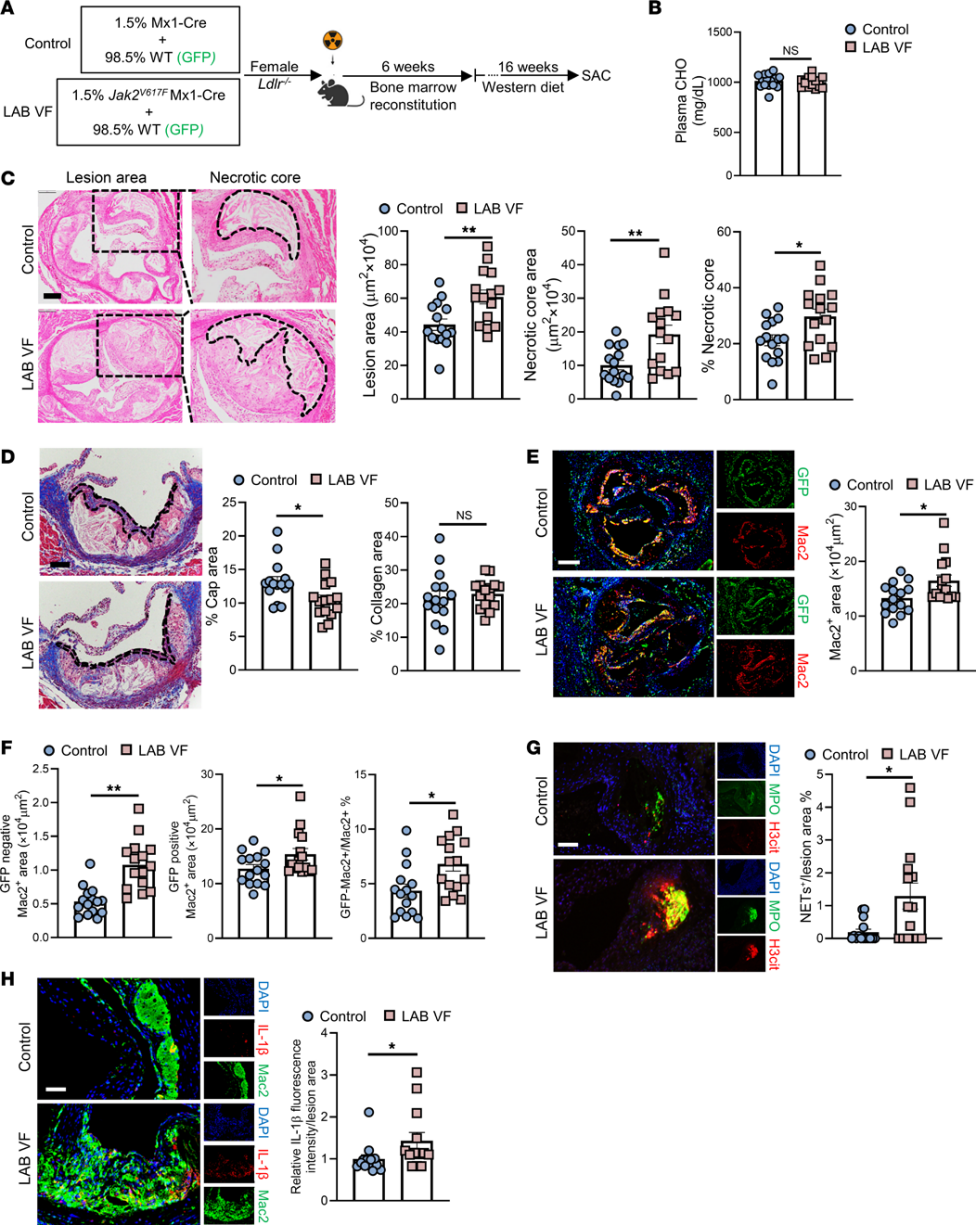
Accelerated atherogenesis and plaque instability in LAB *Jak2^VF^* CH mice. (**A**) Experimental design. (**B**) Plasma cholesterol (CHO) levels. (**C**) Representative H&E staining and quantification of the lesion and necrotic core areas in aortic root sections. Necrotic core regions are indicated by dotted lines. Scale bar: 200 μm. Original magnification, ×100. (**D**) Aortic root sections were stained with Masson’s trichrome for the fibrous cap (outlined by dashed lines) and collagen content area and then quantified as the ratio of the total lesion area. Scale bar: 100 μm. (**E** and **F**) Representative immunofluorescence staining of macrophages (anti-Mac2) and GFP and quantification of the absolute Mac2^+^ area, the GFP^–^Mac2^+^ (Mx1-Cre or *Jak2^VF^* macrophages) area, and the GFP^+^Mac2^+^ area (WT macrophages), as well as the GFP^–^Mac2^+^ area as the percentage of the total Mac2^+^ area in aortic root sections. Scale bar: 250 μm. Original magnification, ×50. (**G**) Representative immunofluorescence staining for H3cit and MPO and quantification of the double-positive area (NETosis) as a percentage of the lesion area. Scale bar: 200 μm. Original magnification, ×200. (**H**) Representative immunofluorescence staining for IL-1β and anti-Mac2 (macrophages) and quantification of the fluorescence intensity of IL-1β were performed and normalized to the lesion area. Scale bar: 50 μm. Original magnification, ×100. Data are presented as the mean ± SEM. *n* = 15 (**B**–**G**), *n* = 13 (**H**). *P < 0.05 and **P < 0.01, by unpaired, 2-tailed Student’s *t* test.

We recently developed a LAB model of *Jak2^VF^* CH by mixing 1.5% *Jak2^VF^* bone marrow (BM) with 98.5% WT BM and transplantation into LDL receptor–deficient (*Ldlr^–/–^*) mice. These mice displayed accelerated FeCl_3_-stimulated carotid artery thrombosis (25). To assess the effect of LAB *Jak2^VF^* CH on atherosclerosis, we transplanted 1.5% *Jak2^VF^* mixed with 98.5% GFP^+^ WT BM cells into lethally irradiated *Ldlr^–/–^* female mice (Figure 1A). After recovery, mice were fed a Western diet (WD) for 16 weeks to promote advanced atherosclerotic lesions (Figure 1A). Similar to an earlier study (25), we found that blood cell counts were unchanged, and there was no expansion of *Jak2^VF^* alleles within WBCs, neutrophils, monocytes, or monocyte subsets (Supplemental Figure 1, A and B; supplemental material available online with this article; https://doi.org/10.1172/JCI182939DS1). In striking contrast to higher VAF mice (34), plasma cholesterol levels were identical between the groups (Figure 1B), probably reflecting the lack of splenomegaly that accelerates LDL degradation in MPNs (35). The atherosclerotic lesion area in the LAB *Jak2^VF^* CH group was increased by approximately 50% and the necrotic core area by nearly 2-fold (Figure 1C), with size effects similar to those seen in the 20% *Jak2^VF^* CH mice (34). Moreover, the area of the fibrous cap, which is considered a protective structure in human plaques (36), was decreased in the LAB mice (Figure 1D), while there was comparable lesional collagen content between the groups (Figure 1D). We observed increased macrophage staining areas in lesions, including in both GFP^+^ (WT) and GFP^–^ (*Jak2^VF^*) macrophages, in LAB *Jak2^VF^* CH mice compared with controls, along with an increased ratio of mutant/WT macrophages in the lesions (Figure 1, E and F). We also observed a moderate expansion of GFP^–^ (*Jak2^VF^*) cells in LAB mice compared with controls, but they still only represented approximately 7% of total macrophages in the lesions (Figure 1F). Studies have shown increased neutrophil extracellular traps (NETs) in lesions as a result of aggravated inflammation (37–39). As assessed by costaining of citrullinated histones (3H-Cit) and myeloperoxidase (MPO), we found increased NETs in the LAB *Jak2^VF^* CH lesions compared with control mice (Figure 1G). These data showed a marked increase in atherosclerosis with features of plaque destabilization in LAB *Jak2^VF^* CH mice, involving both mutant and WT macrophages. Since a minor population of *Jak2^VF^* macrophages was inducing these overall changes, there was likely major inflammatory crosstalk from mutant to WT cells in lesions.

Next, we attempted to identify inflammatory cytokines responsible for the crosstalk. IL-1β is an apical cytokine that plays an important role in human CVD (5), and we have shown that IL-1β and AIM2 inflammasome–induced pyroptosis promote features of plaque instability in higher VAF *Jak2^VF^* CH mice (34). Immunostaining revealed an increased abundance of IL-1β in LAB mice compared with control mice (Figure 1H). We noted an increase in *Il1b* mRNA expression in isolated aortic macrophages from LAB mice in both GFP^+^ and GFP^–^ cells (Supplemental Figure 1C), consistent with IL-1 induction of its own expression (40) in both *Jak2^VF^* and WT macrophages. A survey of other potential inflammatory mediators showed trends for increased *Il6*, *Tnfa*, and *Ifng* expression in macrophages from LAB lesions compared with control lesions (Supplemental Figure 1D). Immunostaining confirmed a significant increase of IL-6 staining, which may be increased downstream of IL-1 signaling (41, 42) in LAB mice, but not of TNF-α or IFN-γ staining (Supplemental Figure 1, E–G).

### Inflammatory crosstalk is mediated through IL-1 receptor, type 1–dependent signaling.

**Figure 2:**
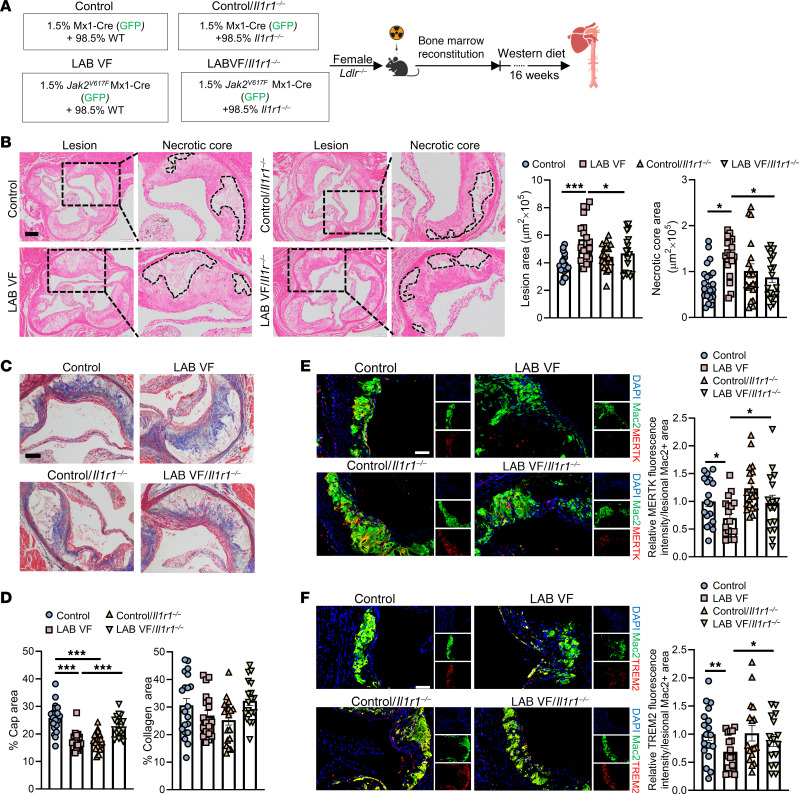
Accelerated atherogenesis in LAB mice is reversed by IL-1R1 deficiency in non-*Jak2^VF^* cells. (**A**) Experimental design. (**B**) Representative H&E staining and quantification of the lesion and necrotic core areas in aortic root sections. Necrotic core regions are indicated by dashed lines. Scale bar: 200 μm. Original magnification, ×100. (**C** and **D**) Aortic root sections were stained with Masson’s trichrome for the fibrous cap and collagen content area and quantified as the ratio of total lesion area. Scale bar: 100 μm. Original magnification, ×200. (**E** and **F**) Representative immunofluorescence staining for MERTK (**E**) or TREM2 (**F**) and anti-Mac2 (macrophages). The fluorescence intensity of MERTK (**E**) and TREM2 (**F**) was quantified and normalized by the lesional macrophage area. Scale bars: 50 μm. Original magnification, ×200. Data are presented as the mean ± SEM. *n* = 20 (control), *n* = 19 (LAB VF), *n* = 19 (control/*II1r^–/–^*), *n* = 18 (LAB VF/*II1r^–/–^*) (**B** and **D**); *n* = 18 (control), *n* = 18 (LAB VF), *n* = 19 (control/*II1r^–/–^*), *n* = 16 (LAB VF/*II1r^–/–^*) (**E**); *n* = 18 (control), *n* = 17 (LAB VF),*n* = 17 ( control/*II1r^–/–^*), *n* = 16 (LAB VF/*II1r^–/–^*) (**F**). *P < 0.05, **P < 0.01, and ***P < 0.001, by 2-way ANOVA.

To test the hypothesis that inflammatory crosstalk from mutant to WT cells is mediated through IL-1 signaling, we used IL-1 receptor 1 deficiency (*Il1r1^–/–^*) in non-*Jak2^VF^* BM cells in the LAB mice (Figure 2A). Donor BM in this cohort included control or *Jak2^VF^* (GFP^+^) BM (1.5%, tagged with GFP to better identify mutant cells) mixed with WT or *Il1r1^–/–^* BM (98.5%, GFP^–^) that was transplanted into irradiated *Ldlr^–/–^* mice (Figure 2A). After 6 weeks of recovery from BM transplantation (BMT), mice were placed on the WD for 16 weeks. At the end of WD feeding, the 4 groups had similar circulating WBCs, RBCs, and platelets (Supplemental Figure 2A). We also observed comparable GFP^+^ WBCs among groups as well as body weight, spleen weight, and liver weight (Supplemental Figure 2, B and C).

Deficiency of IL-1R1 in non-*Jak2^VF^* cells reversed the increase in plaque size and necrotic core area in LAB *Jak2^VF^* mice but did not affect these parameters in control mice (Figure 2B). The reduced fibrous cap in LAB *Jak2^VF^* mice was partially reversed by IL-1R deficiency (Figure 2C). Consistent with prior reports (43), IL-1R deficiency reduced fibrous cap formation in control mice lacking the *Jak2^VF^* mutation (Figure 2, C and D). Collagen content showed no significant change in LAB *Jak2^VF^* mice with or without IL-1R deficiency (Figure 2, C and D).

Impaired efferocytosis has been shown to promote necrotic core formation and decrease fibrous cap thickness in advanced atherosclerotic plaques (44–46). We thus assessed the expression of the phagocytic receptors MERTK and TREM2, which have both been implicated in efferocytosis and necrotic core formation in atherosclerosis (27, 47). Immunostaining revealed an approximate 30% reduction in receptor levels in lesions of LAB mice (Figure 2, E and F). However, this change was reversed by IL-1R1 deficiency in non-*Jak2^VF^* cells, leading to a significant approximately 1.4-fold increase in MERTK levels and a significant approximately 1.3-fold increase in TREM2 levels (Figure 2, E and F). The role of these receptors in plaque development was further assessed with in vivo models (see below).

### IL-1–mediated crosstalk in myeloid cells induces pyroptosis and NETosis.

**Figure 3:**
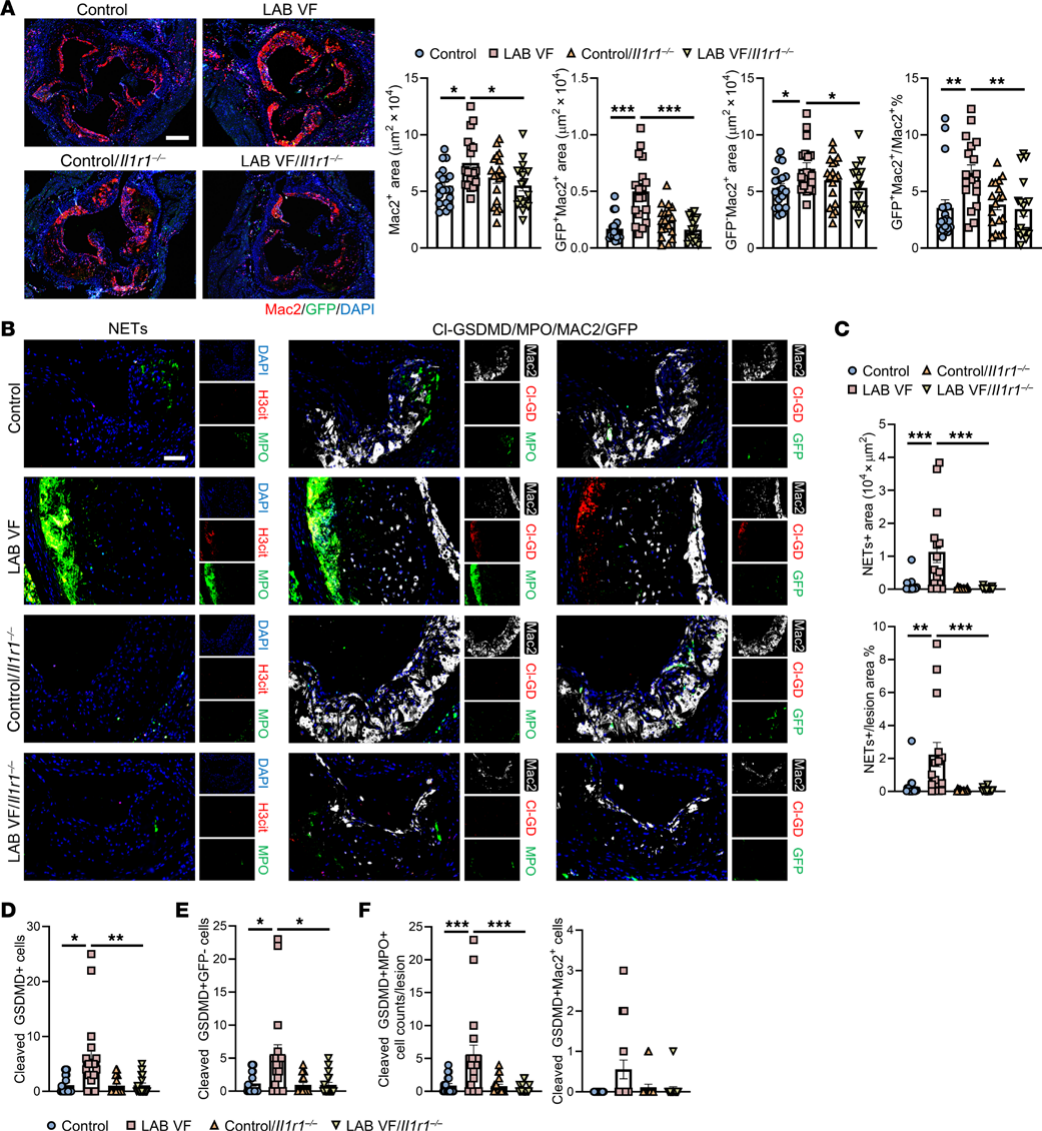
Increased macrophage infiltration, NETosis, and neutrophil inflammasome activation in lesions of LAB mice, all of which are reversed by IL-1R deficiency in non-*Jak2^VF^* cells. (**A**) Representative immunofluorescence staining for anti-Mac2 (macrophages) and GFP, and quantification of the absolute Mac2^+^ area, GFP^+^Mac2^+^ (Mx1-Cre or *Jak2^VF^* macrophages) area, GFP^–^Mac2^+^ area, and GFP^+^Mac2^+^ area as the percentage of the total Mac2^+^ area in aortic root sections. Scale bar: 250 μm. Original magnification, ×50. (**B**) Representative immunofluorescence staining for H3cit and MPO (NETosis), cleaved gasdermin D, anti-MPO (neutrophils), anti-Mac2 (macrophages), and GFP in the lesions. Scale bar: 100 μm. Original magnification, ×50. (**C**) Quantification of absolute area of NETosis^+^ area and as the percentage of the lesion area. (**D**) Quantification of cleaved GSDMD^+^ cell counts in the aortic root lesions. (**E**) Quantification of cleaved-GSDMD^+^GFP^–^ cell counts in the aortic root lesions. (**F**) Quantification of cleaved-GSDMD^+^MPO^+^ or Mac2^+^ cell counts in the aortic root lesions. Data are presented as the mean ± SEM. *n* = 19 (control), *n* = 18 (LAB VF), *n*=18 (control/*II1r^–/–^*), *n* = 17 (LAB VF/*II1r^–/–^*) (**A**); *n* = 18 (control), *n* = 15 (LAB VF), *n* = 16 (control/*II1r^–/–^*), *n* = 17 (LAB VF/*II1r^–/–^*) (**C**); *n* = 18 (control), *n* = 18 (LAB VF), *n* = 18 (control/*II1r^–/–^*), *n* = 16 (LAB VF/*II1r^–/–^*) (**D** and **E**); *n* = 18 mice per group (**F**). *P < 0.05, **P < 0.01, and ***P < 0.001, by 2-way ANOVA.

IL-1R1 deficiency reversed the increase in macrophages in LAB mice for both GFP^+^ and GFP^–^ macrophages and decreased GFP^+^ macrophages as a percentage of total macrophages in plaques (Figure 3A), indicating that IL-1 signaling in both *Jak2^VF^*-mutant and non-*Jak2^VF^*-mutant macrophages was increasing their content in plaques. We found that when IL-1R1 was absent in WT cells, elevated NETosis in the LAB lesions was abolished (Figure 3, B and C). To assess pyroptosis, we used an antibody to detect the N-terminal, cleaved form of gasdermin D (Cl-GSDMD), the mediator of pyroptosis downstream of inflammasomes (48). The specificity of this antibody was verified by Western blotting (WB) and by lack of staining in the lesions of *Gsdmd*-deficient mice (Supplemental Figure 2, D and E). However, WB showed that, while the antibody mainly recognized the cleaved form of GSDMD, there was a faint band corresponding to full-length GSDMD (Supplemental Figure 2D). To assess in vivo specificity, we compared immunostaining by this antibody with that of an antibody recognizing full-length GSDMD. In sections from 4 different mice, there was no overlap in staining of the 2 antibodies, suggesting specificity of the Cl-GSDMD antibody (a representative section is shown in Supplemental Figure 2F). The full-length antibody stained primarily in the cellular area and the Cl-GSDMD antibody predominantly in the necrotic core region. This suggests that the Cl-GSDMD antibody mainly recognized cleaved GSDMD in necrotic regions where pyroptosis had occurred. Cl-GSDMD staining was significantly increased in LAB mice, and this appeared to be especially the case in areas of NETosis (Figure 3, B and D). Further characterization revealed that Cl-GSDMD was largely expressed in GFP^–^ neutrophils with lower expression in GFP^–^ macrophages (Figure 3, B, E, and F). This suggests that IL-1 drives pyroptosis in bystander neutrophils, potentially promoting NETosis (49) in LAB mice (Figure 3, B–F).

### Resistance to MERTK cleavage in WT cells reduces plaque necrosis in LAB Jak2^VF^ CH mice.

**Figure 4:**
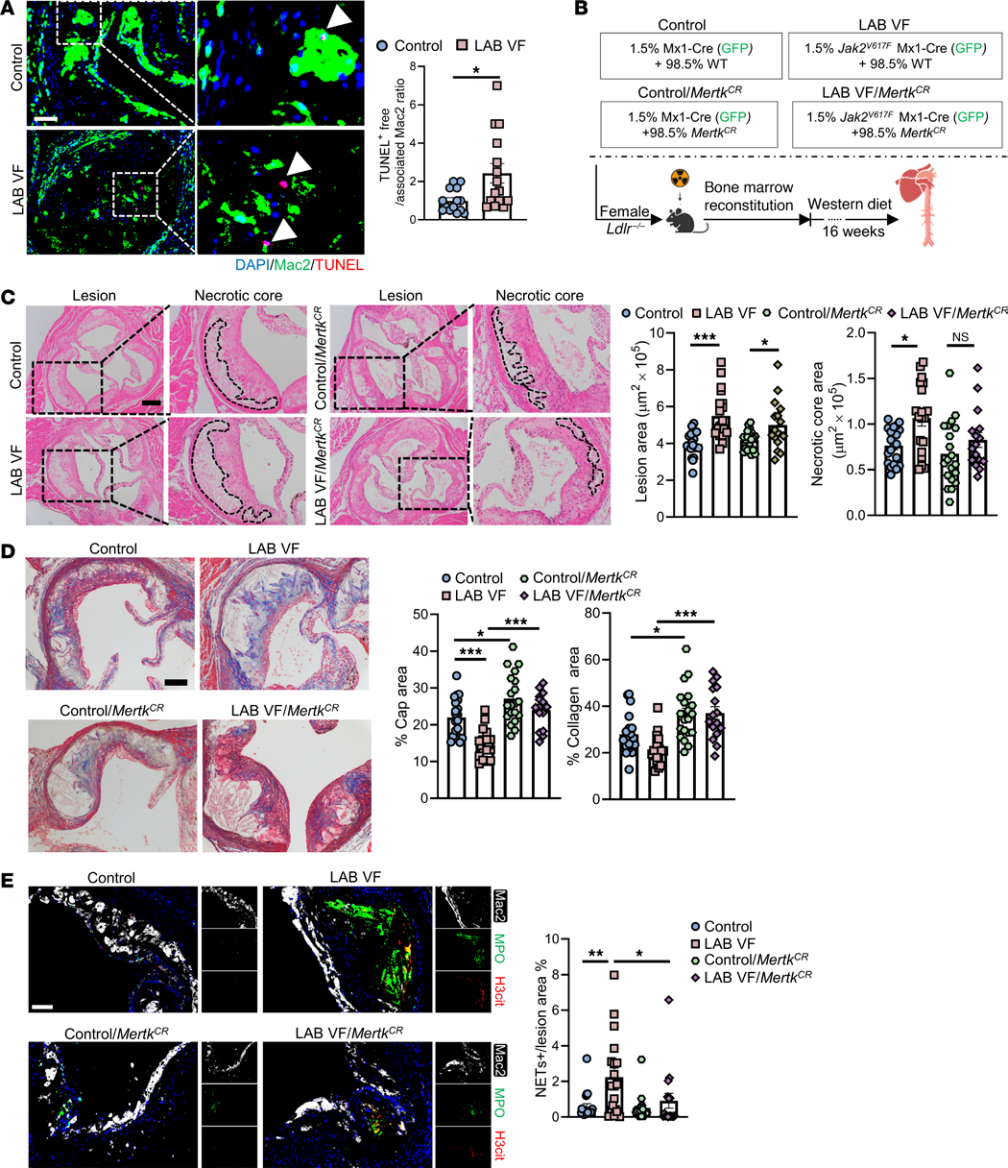
Presence of *Mertk^CR^* cells alleviates plaque necrosis in LAB mice. (**A**) Staining of macrophages, TUNEL and quantification of efferocytosis (ratio of TUNEL^+^-free cells to macrophage-associated TUNEL^+^ cells) in aortic root sections. Arrowheads show TUNEL^+^ cells. Scale bar: 50 μm. Original magnification, ×200 (enlarged insets). (**B**) Experimental design. (**C**) Representative H&E staining and quantification of the lesion area and necrotic core area in aortic root sections. Necrotic core regions are indicated by dashed lines. Scale bar: 200 μm. Original magnification, ×100 (enlarged insets). (**D**) Aortic root sections were stained with Masson’s trichrome for the fibrous cap and collagen content area and quantified as the ratio of the total lesion area. Scale bar: 100 μm. Original magnification, ×200 (enlarged insets). (**E**) Representative immunofluorescence staining for H3cit, MPO (NETosis), and Mac2 and quantification of the ratio of the double-positive area (NETs) area to the lesion area. Scale bar: 50 μm. Original magnification, ×200. *P < 0.05, **P < 0.01, and ***P < 0.001, by 2-way ANOVA. Data are presented as mean ± SEM. *n* =14 (control), *n* = 15 (LAB VF) (**A**); *n* = 20 (control), *n* = 19 (LAB VF), *n* = 20 (control/*Mertk^CR^*), *n* = 16 (LAB VF/*Mertk^CR^*) (**C** and **D**); *n* = 20 (control), *n* = 19 (LAB VF), *n* = 19 (control/*Mertk^CR^*), *n* = 16 (LAB VF/*Mertk^CR^*) (**E**). *P < 0.05, **P < 0.01, and ***P < 0.001, by unpaired, 2-tailed Student’s *t* test (**A**) or 2-way ANOVA (**C** and **E**).

To assess the potential functional effect of reduced macrophage MERTK expression in LAB *Jak2^VF^* mice, we examined in vivo efferocytosis as described previously (50). We found impaired in situ efferocytosis in lesions of *Jak2^VF^* LAB mice (Figure 4A). To evaluate the role of reduced MERTK in lesion development, we used a genetically modified, cleavage-resistant version of the *Mertk* gene (*Mertk^CR^*) (27). *Mertk^CR^* *Ldlr^–/–^* mice have been shown to have increased macrophage efferocytosis and decreased necrotic cores in aortic root lesions (27). *Mertk^CR^* BM (98.5%) was mixed with *Jak2^VF^* or control BM (1.5%) and transplanted into *Ldlr^–/–^* mice (Figure 4B). After 16 weeks of WD feeding, the mice had comparable circulating WBCs, RBCs, and platelets among the groups (Supplemental Figure 3A). We also found unchanged GFP^+^ WBC percentages and spleen weights in the 4 groups (Supplemental Figure 3, B and C). In *Jak2^VF^* LAB mice, administration of *Mertk^CR^* BM eliminated the difference in necrotic core areas between *Jak2^VF^* LAB mice and the control group mice (Figure 4C). We observed no significant change in the overall lesion area (Figure 4C), indicating that the reduction in necrotic core area was not secondary to the reduced plaque size. LAB/*Mertk^CR^* mice also showed an increased cap area and an increased collagen staining area (Figure 4D), indicating improved features of plaque stability that could be related to increased TGF-β1 production in *Mertk^CR^* mice, as previously shown (27). Unexpectedly, we observed that increased NETosis was significantly reduced in LAB *Mertk^CR^* mice (Figure 4E), indicating a role of MERTK in NET formation or clearance in lesions. Together, the results suggest that the aggravation of plaque necrosis and NETosis in LAB mice was mediated at least in part by MERTK cleavage downstream of IL-1β in nonmutant cells in plaques.

**Figure 5:**
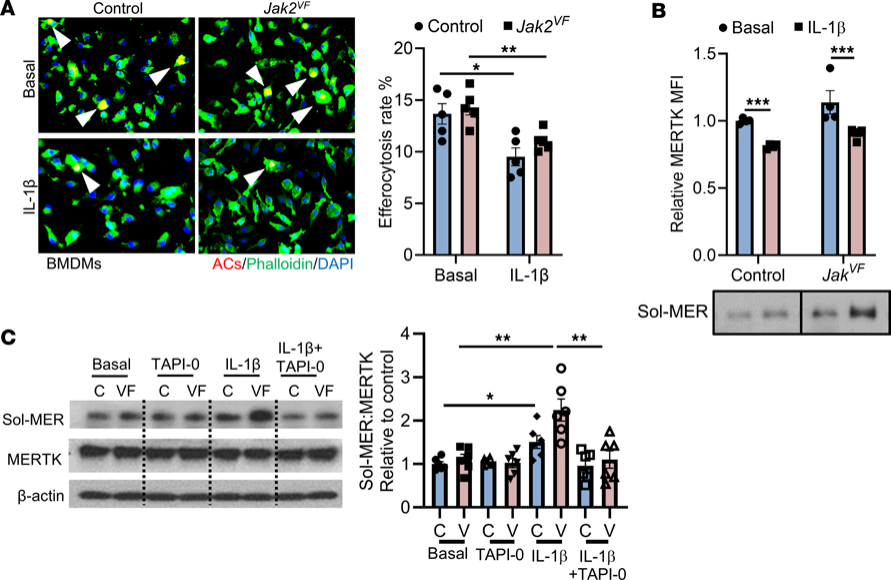
IL-1β promotes MERTK cleavage through activation of P38/ADAM17 signaling. (**A**) BMDMs from Mx1-Cre (control) and *Jak2^VF^* mice were pretreated or not with IL-1β (25 ng/mL) for 6 hours and then incubated with PKH26-labeled ACs (red) for 45 minutes at a 5:1 AC/macrophage ratio. Phalloidin (green) was used to label F-actin in cells. Bar graph shows quantification of the efferocytosis of macrophages (arrowheads) relative to the percentage of total macrophages. *n* = 5 independent experiments. (**B**) BMDMs from Mx1-Cre (control [C]) and *Jak2^VF^* (VF) mice were treated with or not with IL-1β (25 ng/mL) for 6 hours, followed by assays for cell-surface MERTK by flow cytometry and soluble MERTK (Sol-MER) levels in culture media by Western blotting (the lanes were run on the same gel but were noncontiguous). *n* = 4 independent experiments. (**C**) BMDMs were treated or not with the ADAM17 inhibitor TAPI-0 (5 μM) or IL-1β (25 ng/mL) for 6 hours and then assayed for cellular MERTK and soluble MERTK in culture media by Western blotting. *n* = 6 independent experiments. Data are presented as the mean ± SEM. *P < 0.05, **P < 0.01, and ***P < 0.001, by 2-way ANOVA.

To assess the possibility that reduced MERTK might be linked to increased active IL-1β in plaques, we conducted efferocytosis assays in which BM-derived macrophages (BMDMs) were treated with IL-1β for 6 hours and then incubated with PKH26-labeled, UV-induced apoptotic Jurkat cells (ACs). The percentage of macrophages that had internalized ACs was significantly lower in IL-1β–treated macrophages than in nontreated cells, regardless of genotype (Figure 5A). The effect was similar in WT and *Jak2^VF^* cells, since the latter were not treated with inflammasome activators. To determine whether IL-1β signaling regulates MERTK cleavage or if other cytokines may be involved, we treated BMDMs from control and *Jak2^VF^* mice with IL-1β, IL-6, IFN-γ, and IL-1α. We found that IL-1β, but not other cytokines, induced MERTK cleavage, as shown by a decrease in cell-surface MERTK expression and an increase in soluble MERTK in media of both control and *Jak2^VF^* BMDMs, with a more pronounced effect in *Jak2^VF^* cells (Figure 5B and Supplemental Figure 3D). The extracellular domain of MERTK can undergo ADAM17-mediated proteolytic cleavage, and P38 activity is required for ADAM17-mediated MERTK cleavage (51, 52). IL-1β activated P38 in WT macrophages, as reported previously (53), and in *Jak2^VF^* macrophages (Supplemental Figure 3E). Moreover, P38 is known to activate ADAM17 (54, 55). To determine whether ADAM17 is involved in IL-1β–mediated MERTK cleavage, we treated macrophages with the ADAM17 inhibitor TAPI-0 and found that IL-1β–induced MERTK cleavage was blocked (Figure 5C). These findings indicate that IL-1β activated p38 MAPK, which led to ADAM17-mediated cleavage of MERTK, establishing a mechanistic link between inflammasome activation and impaired efferocytosis.

### The TREM2-activating antibody 4D9 increases fibrous cap formation.

**Figure 6:**
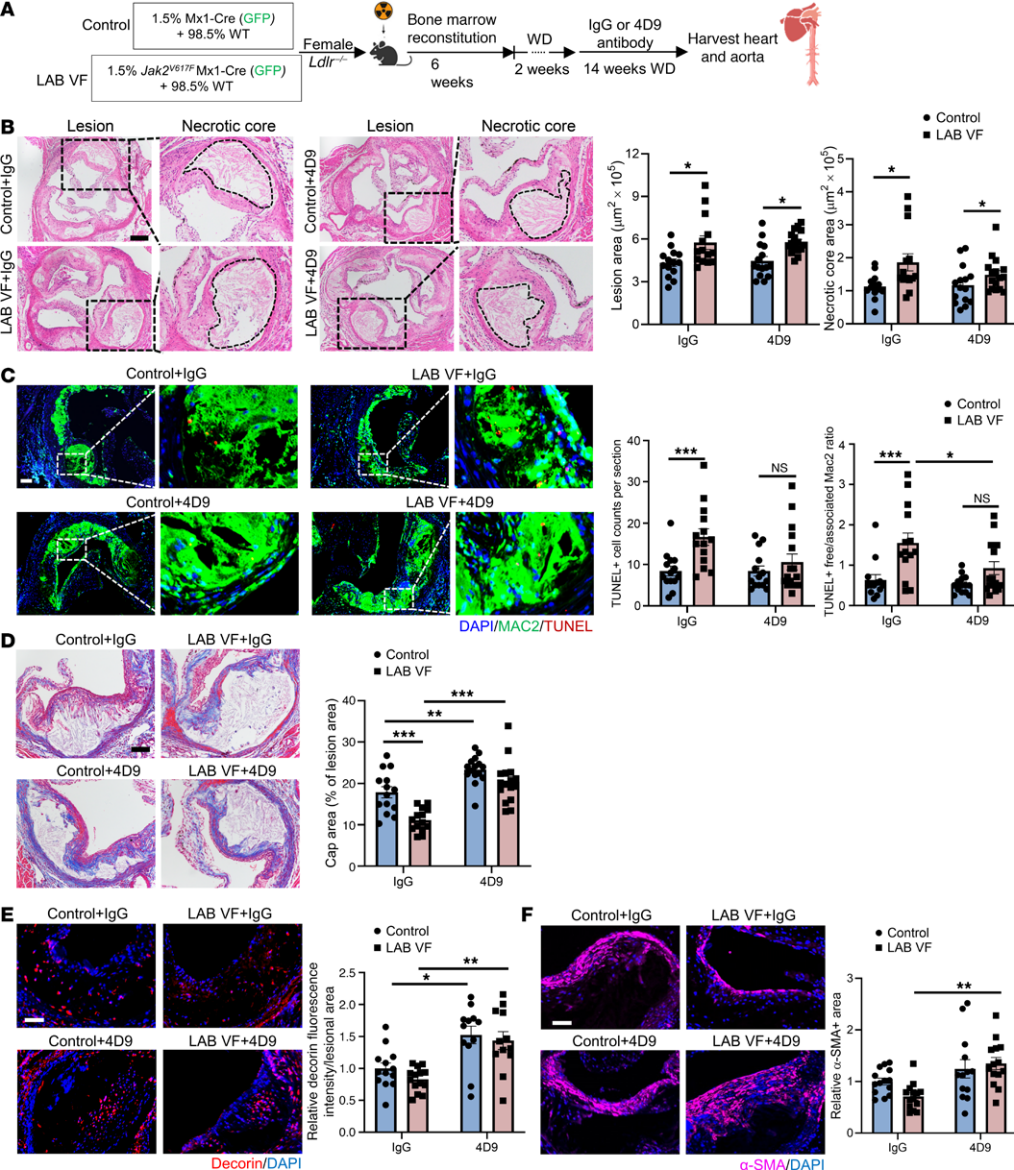
The TREM2 activating antibody 4D9 enhances plaque stability by increasing the thickness of fibrous caps. (**A**) Experimental design. (**B**) Representative H&E staining and quantification of the lesion area and necrotic core area in aortic root sections. Necrotic core regions are indicated by dashed lines. Scale bar: 200 μm. Original magnification, ×100 (enlarged insets). (**C**) Staining of macrophages and for TUNEL and quantification of efferocytosis (ratio of TUNEL^+^-free cells to macrophage-associated TUNEL^+^ cells) in aortic root sections. Scale bar: 50 μm. Original magnification, ×100 (enlarged insets). (**D**) Aortic root sections were stained with Masson’s trichrome for the fibrous cap and quantified as the ratio of the total lesion area. Scale bar: 75 μm. Original magnification, ×200 (enlarged insets). (**E**) Representative immunofluorescence staining for decorin and quantification of the fluorescence intensity of decorin were performed and normalized to the lesion area. Scale bar: 50 μm. Original magnification, ×200 (enlarged insets). (**F**) Immunofluorescence staining for α-SMA and quantification of the relative α-SMA^+^ area in lesions. Scale bar: 50 μm. Data are presented as the mean ± SEM. *n* = 14 (control+IgG), *n* = 14 (LAB VF+IgG), : *n* = 15 (control+4D9), *n* = 1 (LAB VF+4D9) (**B** and **D**); *n* = 13 (control+IgG), *n* = 12 (LAB VF+IgG), *n* = 14 (control+4D9), *n* = 12 (LAB VF+4D9) (**E**); *n* = 14 (control+IgG), *n* = 13 (LAB VF+IgG), *n* = 14 (control+4D9), *n* = 14 (LAB VF+4D9) (**F**). *P < 0.05, **P < 0.01, and ***P < 0.001, by 2-way ANOVA.

We next assessed the in vivo role of TREM2 in plaque stabilization in LAB mice using the TREM2-activating antibody 4D9 (56). 4D9 elicits a major increase in cell-surface TREM2 and TREM2 signaling and concomitantly reduces proteolytic shedding of TREM2 (56). To determine if activation of TREM2 could improve atherosclerosis in LAB *Jak2^VF^* CH mice, we administered 4D9 or isotype control IgG antibodies by i.p. injection beginning 2 weeks after initiation of the WD and continued the injections and the diet for an additional 14 weeks (Figure 6A). The antibody treatment did not alter the allele burden or blood cell counts in the LAB mice (Supplemental Figure 4A). 4D9 treatment significantly increased TREM2 expression in plaque myeloid cells isolated by flow cytometry (*P* = 0.003 for group effect, by 2-way ANOVA), bringing TREM2 expression to similar levels in both LAB mice and controls, and showing efficacy of the antibody (Supplemental Figure 4B).

Analysis of lesions in the proximal aorta showed no change in lesion area (Figure 6B). The necrotic core area was reduced by approximately 20%, but the change was not significant (Figure 6B). Even though the necrotic core area trended toward reduction, TUNEL^+^ cells were reduced by 4D9 treatment, and the impaired efferocytosis in *Jak2^VF^* lesions was significantly improved by 4D9 (Figure 6C). 4D9 produced a marked increase in cap area in both control (~1.3-fold) and LAB *Jak2^VF^* CH (~1.8-fold) mice in response to 4D9 treatment, eliminating the difference between the groups (Figure 6D). This was associated with an increase in intimal staining for both decorin (control: 1.52-fold vs. LAB VF: 1.72-fold) and α smooth muscle actin (α-SMA) (control: 1.25-fold vs. LAB VF 1.90-fold), which are potential markers for fibroblasts and smooth muscle cells (SMCs), respectively (Figure 6, E and F). Thus, 4D9 treatment led to improvements in parameters associated with plaque destabilization in both control and *Jak2^VF^* CH mice, with the most dramatic effects on the fibrous cap area and a numerically larger effect in *Jak2^VF^* LAB mice than in controls.

**Figure 7:**
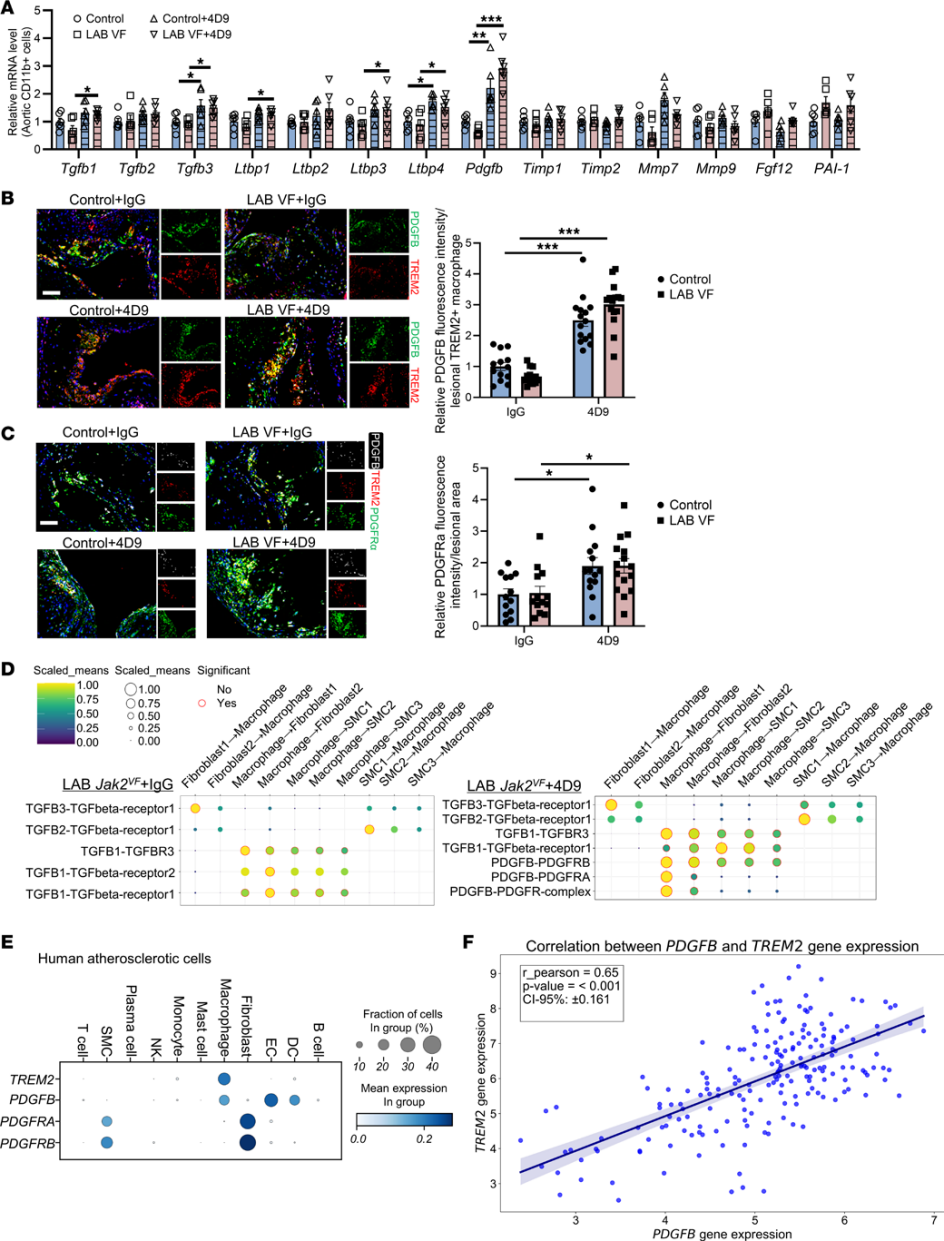
4D9-mediated TREM2 activation promotes fibrogenesis through macrophage *Pdgfb*–fibroblast *Pdgfra* crosstalk. (**A**) Relative mRNA of fibrogenic genes in isolated aortic CD11b^+^ cells in mice. *n* = 6, each replicate represents pooled digested aortic cells from 2–3 mice. (**B**) Representative immunofluorescence staining for PDGFB, TREM2, and anti-Mac2 (macrophages). The fluorescence intensity of PDGFB was quantified and normalized by the lesional TREM2^+^ macrophage area. Scale bar: 50 μm. Original magnification, ×200 (enlarged insets). (**C**) Representative immunofluorescence staining for TREM2, PDGFB, and PDGFRa. The fluorescence intensity of PDGFRa was quantified and normalized by the lesion area. Scale bar: 50 μm. Original magnification, ×200 (enlarged insets). (**D**) Dot plot of ligand-receptor interactions between macrophage and SMC/fibroblast subpopulations. Ligand and cognate receptors are shown on the *y* axis. Circle size denotes the *P* value (permutation test); color (yellow, high; blue, low) denotes the average ligand and receptor expression levels in interacting subpopulations. (**E**) scRNA-Seq analysis of human atherosclerotic tissue shows the distribution of TREM2, PDGFB, PDGFRA, and PDGFRB transcripts in all major vascular cell types. (**F**) Pearson’s correlation analysis was performed on 202 atherosclerotic tissue samples and shows the correlation between TREM2 and PDGFB. Data are presented as the mean ± SEM. *n* = 13 (control+IgG), *n* = 13 (LAB VF+IgG), *n* = 14 (control+4D9), *n* = 15 (LAB VF+4D9) (**B** and **C**). *P < 0.05 and ***P < 0.001, by 2-way ANOVA (**A** and **C**).

To explore the underlying mechanisms of the fibrogenic effects of 4D9, we isolated aortic CD11b^+^ cells and screened for TREM2-related fibrogenic mediators (57). This screen included *Tgfb* family members (*Tgfb1*, *Tgfb2*, *Tgfb3*), latent TGF-β–binding proteins (*Ltbp1–4*), PDGFB (*Pdgfb*), tissue inhibitors of matrix metalloproteases (*Timp*, *Timp2*) (58), matrix metalloproteases (*Mmp7*, *Mmp9*), FGF12 (*Fgf12*) (59), and plasminogen activators (*PAI-1*) (60) (Figure 7A). The screen showed modest, less than 2-fold increases in *Tgfb*-related genes in response to 4D9 treatment (Figure 7A). In contrast, *Pdgfb* mRNA was increased 3- to 5-fold by 4D9 treatment, with significant increases in both control and LAB cells (Figure 7A). 4D9 increased lesional PDGFB levels by 2-fold compared with IgG (Supplemental Figure 4E). Moreover, PDGFB^+^TREM2^+^ double-positive macrophages were increased by 2- to 4-fold in 4D9-treated mice (Figure 7B) and were almost exclusively found in close proximity to the cap region (Figure 7B). We also stained lesions for PDGFRα, a PDGF receptor that is specifically expressed on fibroblasts (61). We observed a significant, 2-fold increase in PDGFRα staining in both control and *Jak2^VF^* LAB groups in response to 4D9 treatment (Figure 7C), indicating increased numbers of fibroblasts. 4D9 slightly increased TGF-β1 staining in lesions (*P* = 0.02 for treatment effect, by 2-way ANOVA) (Supplemental Figure 4E).

To define the response to 4D9 on a cellular level, we conducted single-cell RNA-Seq (scRNA-Seq) analysis on whole aortas from LAB mice treated with 4D9 or control IgG. Similar to previous reports (34, 62), scRNA-Seq analysis revealed 13 cell populations, including SMCs, fibroblast-like cells, and macrophages (Supplemental Figure 5A). Feature plots revealed that *Trem2* was predominantly expressed in macrophages; *Pdgfb* was expressed in monocytes and macrophages as well as other cell types (Supplemental Figure 5, A and B); *Pdgfra* was primarily found in fibroblasts (Supplemental Figure 5, A and B); and *Tgfb1* was widely expressed in all cell types (Supplemental Figure 5, A and B). To assess potential ligand-receptor interactions between macrophages, SMCs, and fibroblasts based on the scRNA-Seq data, we used Cellphone-DB, a computational framework that can be used to evaluate cell-cell communication mediated by a large variety of ligand-receptor complexes (63). Functional analyses using CellPhone-DB suggested that in both IgG- and 4D9-treated LAB mice, macrophages showed signaling between TGF-β and its cognate receptor TGF-βR on SMCs and fibroblasts (Figure 7D). However, 4D9 treatment specifically increased macrophage PDGFB signaling to fibroblast PDGFRa and PDGFRb (Figure 7D), consistent with the immunostaining and fibrogenic mediator gene screening data.

In order to determine the effect of TREM2 activation on MERTK expression, we stained the aortic root section and found that reduced MERTK expression in LAB mice was unaffected by 4D9 treatment (Supplemental Figure 4F). Flow cytometry confirmed a decrease in MERTK expression on aortic macrophages, with no effect of 4D9 treatment (Supplemental Figure 4G). In accord with our in vivo results, the surface expression of MERTK on BMDMs was also unaffected by 4D9 (Supplemental Figure 4H). We isolated aortic CD11b^+^ cells to determine if resistance to the MERTK cleavage may also affect *Pdgfb* expression, however, quantitative PCR (qPCR) analysis revealed no change in the mRNA expression level (Supplemental Figure 4I). Our data suggest that TREM2 and MERTK may have acted independently in LAB mice to promote different features of plaque instability. 4D9-mediated TREM2 activation primarily improved atherosclerosis in LAB *Jak2^VF^* by activating a fibrogenic pathway, possibly mediated by macrophage *Pdgfb*/fibroblast *Pdgfra* signaling.

### TREM2 and PDGFB in human carotid plaque samples.

Analysis of scRNA-Seq data from human advanced carotid plaques revealed that both PDGFB and TREM2 were highly expressed in macrophages (Figure 7E). Similar to our findings in mice, human plaque fibroblasts showed high expression of *PDGFRA* (Figure 7E). Consistent with the effects the TREM2-agonizing antibody in mice, analysis of bulk RNA-Seq data from 202 human atherosclerotic plaque specimens revealed a significant positive correlation between the expression of *PDGFB* and *TREM2* mRNA (Figure 7F).

### 4D9 increases PDGFB expression via a Syk/AKT/β-catenin–dependent pathway.

Next, we sought to understand how 4D9 treatment in macrophage induces PDGFB expression. We observed a significant increase in PDGFB levels in control and *Jak2^VF^* BMDMs treated with 4D9 (Supplemental Figure 6A), demonstrating a cell-autonomous effect. 4D9 treatment increased *Pdgfb* mRNA levels in a time-dependent fashion (Supplemental Figure 6B). Moreover, *Pdgfb* mRNA expression was markedly reduced in *Trem2*-KO macrophages, and, as expected, we found no effect of 4D9 treatment in these cells (Supplemental Figure 6B), showing specificity.

4D9 has been shown to activate Syk/AKT downstream of TREM2 (56). 4D9 increased phosphorylated Syk (p-Syk) and p-AKT in both control and *Jak2^VF^* BMDMs (Supplemental Figure 6C). Previous studies have shown that AKT-mediated β-catenin phosphorylation may increase the transcriptional activity of AKT and promote the expression and production of PDGFB in endothelial cells (64). To test this, we treated BMDMs with IgG or 4D9 and found that 4D9 treatment increased β-catenin phosphorylation (Supplemental Figure 6D), in association with a rise in nuclear β-catenin (Supplemental Figure 6E).

To further assess the function of Syk/AKT/β-catenin signaling in the regulation of *Pdgfb* expression in macrophages, we used a selective Syk inhibitor, fostamatinib (65). Pretreatment of BMDMs with fostamatinib abolished increased β-catenin phosphorylation by 4D9 (Supplemental Figure 6F), in parallel with a reversal of 4D9-mediated upregulation of Syk and AKT phosphorylation (Supplemental Figure 6F). Fostamatinib also abolished the increase in nuclear β-catenin levels after 4D9 treatment (Supplemental Figure 6G). In parallel, 4D9-induced upregulation of *Pdgfb* mRNA and protein in BMDMs was reversed by fostamatinib (Supplemental Figure 6, H and I), demonstrating the key role of the Syk signaling pathway in mediating *Pdgfb* upregulation by 4D9.

## Discussion

Our study shows a major role of IL-1–mediated crosstalk from a small number of mutant *Jak2^VF^* macrophages to WT macrophages and neutrophils in the promotion of atherosclerosis and features of plaque destabilization. These changes were mechanistically linked to increased cleavage of macrophage MERTK and TREM2 downstream of elevated IL-1 signaling. Therapeutic approaches to stabilize these receptors and increase their signaling resulted in markedly improved plaque stability, especially in the context of CH. While the noncleavable form of MERTK predominantly reduced necrotic cores, TREM2 activation produced a major effect on the fibrous cap area in both control and LAB mice. We discovered that macrophage PDGFB expression was strongly induced by TREM2 activation and that a macrophage PDGFB/fibroblast PDGFRA pathway may promote the accumulation of fibroblast-like cells in the fibrous cap. Thus, PDGFB signaling that has traditionally been assigned a pro-atherogenic role, in these circumstances may promote plaque stabilization.

The significant effect of clonal hematopoiesis on atherosclerosis in humans and in mouse models has suggested the possibility that inflammatory crosstalk from mutant to WT cells might be involved in the promotion of atherosclerosis. Studies from our and other laboratories have indicated a role of inflammasomes and IL-1β in promoting atherosclerosis in *Jak2^VF^* and *Tet2* CH mouse models, suggesting that IL-1 signaling might have a key role in mediating inflammatory crosstalk (34, 66). This hypothesis was directly supported in the present study, in which a small burden of mutant *Jak2^VF^* cells (1.5%) with only modest expansion in blood and plaques induced prominent atherosclerosis, with reversal of increased lesion and necrotic core areas and fibrous cap thinning by deficiency of the IL-1 receptor in WT cells. A notable finding was the complete loss of NETs due to IL-1R1 deficiency, correlating with a disappearance of cleaved gasdermin D in MPO^+^ cells in the NET staining region of plaques and consistent with the role of neutrophil gasdermin D in the formation of NETs (67). Together with earlier studies (49, 68), our findings provide in vivo evidence that IL-1 secreted by macrophages may induce NETosis via inflammasome activation and pyroptosis of neutrophils (49). While we focused on IL-1, it is possible that other inflammatory factors are involved in crosstalk. For example, Cobo et al. (69) have implicated inflammatory crosstalk mediated by type 1 IFNs in the promotion of CVD in both *TET2* and *DNMT3A* CH. The possibility that DNA damage responses underlie both inflammasome activation and the type 1 IFN response in CH warrants further investigation.

Phagocytic receptors such as MERTK and TREM2 are thought to have an important role in the resolution of inflammatory processes in atherosclerosis. MERTK has been well investigated and shown to promote efferocytosis and increase fibrous cap formation in advanced atherosclerosis (27). These effects have been linked to increased expression of TGF-β that occurs during efferocytosis. Our studies using *Mertk^CR^* mice have provided a mechanistic link between inflammasome activation, IL-1 release, and MERTK cleavage. Studies in BMDMs revealed that IL-1β reduces MERTK levels via activation of p38 map kinase and ADAM17. A similar mechanism has been implicated in the cleavage of TREM2 downstream of IL-1 signaling in NASH (70). These findings suggest a common mechanism to link inflammasome activation to cleavage of MERTK and TREM2 but with distinct outcomes for each receptor. Accordingly, our in vivo studies showed a more significant effect of *Mertk^CR^* on the necrotic core in LAB *Jak2^VF^* mice than in controls, correlating with higher levels of inflammasome activation and NETosis. Unexpectedly, increased NETs in LAB mice were largely reversed by *Mertk^CR^*, suggesting an unexplored role of MERTK in the clearance or formation of NETs. Interestingly, macrophages can phagocytose NETs in an immunologically silent fashion (71), and our data suggest this could depend on MERTK. Since NETosis has been strongly linked to necrotic core formation in mice and humans (72), reduced NETs may also contribute to a reduced necrotic core in LAB mice with IL-1R deficiency or *Mertk^CR^*.

The TREM2 receptor promotes mitochondrial metabolism and proliferation of microglia (73, 74). Targeting TREM2 has emerged as a therapeutic approach to the treatment of Alzheimer’s disease (74, 75). Although TREM2 has been characterized as an efferocytotic receptor (76) compared with *Mertk^CR^*, we found distinct effects of TREM2-activating antibodies in atherosclerotic plaques, with a marked increase in fibrous cap formation and no significant effect on necrotic core formation. Mechanistic studies indicated a key role of TREM2 expression and signaling to increase macrophage *Pdgfb* expression, which could contribute to increased fibrous cap formation in 4D9-treated mice (32). However, the lack of effect of 4D9 on the necrotic core area and MERTK levels suggests that the signaling downstream of *Trem2* via the Syk/AKT/β-catenin pathway that increases *Pdgfb* may be more important in determining plaque features than the potential effects of *Trem2* on liver X receptor (LXR) activation and *Mertk* expression (32).

Traditionally, PDGF was thought to promote atherosclerosis by increasing SMC migration into the intima in injury models and by increasing SMCs and extracellular matrix in atherosclerotic plaques (77). Increased PDGF signaling via SMC *Pdgfrb* in *Lrp1*-KO mice increased aortic atherosclerosis (78), and loss-of-function PDGFD variants are associated with a reduced risk of myocardial infarction (79). Contemporary studies have emphasized the importance of fibrous cap formation in preventing plaque rupture (80, 81), and there may be different outcomes for different PDGF isoforms and receptors. Deletion of *Pdgfb* in hematopoietic chimeras in *Apoe^–/–^* mice markedly delayed fibrous cap formation without reducing the lesion area (82), an antibody against PDGFB reduced SMCs in the intima and cap region (83), and deletion of *Pdgfrb* in SMCs reduced fibromyocytes in plaques and decreased cap thickness (84). Consistent with these studies, our findings using 4D9 show that increased PDGFB in Trem2^Hi^ macrophages associated with increased fibrous cap thickness and increased staining for SMCs and fibroblasts. Our study suggesting that the mechanism of increased fibrous cap formation may involve recruitment of PDGFRα-positive fibroblasts to the fibrous cap is consistent with other recent studies showing that inhibition of IL-1β in *Jak2^VF^* and *Tet2* CH promotes the accumulation of fibroblast-like cells in plaques (62). *Pdgfra* was found to be highly expressed in fibrogenic fibroblast populations that were induced by IL-1β antibodies in both *Jak2^VF^* and *Tet2* CH mice (62). Together, these studies suggest that a signaling axis involving *Pdgfb* in macrophages and fibroblast *Pdgfra* could be important in promoting fibroblast proliferation and migration into the fibrous cap.

A recent study has indicated that TREM2 deficiency promoted necrotic core formation, whereas agonistic TREM antibody 4D9 reduced necrotic formation in early atherosclerotic lesions, but did not report the results of the effect of 4D9 on fibrous cap formation (47). However, necrotic core formation in advanced lesions was unaffected by TREM2 deficiency (47), which is consistent with our finding that TREM2 activation did not significantly improve the necrotic core area after 16 weeks on a WD (47). Van Lengerich et al. reported that a different mAb (AL002a) against the extracellular domain of TREM2 produced a marked increase in fibrous cap and collagen content and a decrease in the necrotic core area of atherosclerotic lesions in high-fat-diet–fed *Ldlr^–/–^* mice (85). While there was an increase in Oil Red O staining of lesions (85), we found no increase in lesion area in the H&E-stained sections of paraffin-embedded lesions. The increased staining with a lipophilic dye could be consistent with accumulation of noninflammatory, TREM2^Hi^ foamy macrophages. A key difference between our studies and these earlier reports is that we assessed the effect of 4D9 in the setting of exaggerated plaque inflammation and reduced TREM2 expression. Although the directionality of 4D9 effects was similar in control mice and LAB mice, the effect appeared to be greater in the LAB mice. This is consistent with the greater role of inflammasome activation and IL-1β secretion in plaque instability features in CH mice compared with control *Ldlr^–/–^* mice (86). Overall, the findings from our and other groups suggest a potential therapeutic benefit of TREM2-agonizing antibodies by stabilization of atherosclerotic plaques.

Our studies in human carotid plaques showed a strong correlation between *PDGFB* mRNA levels and *TREM2* levels and colocalization in macrophages, suggesting human relevance of the findings in mice. Although the clinical significance of LAB CH in atherosclerosis needs to be further clarified, inflammasome activation is prominent in TET2, JAK2^VF^, and probably ASXL1 CH (50, 66, 87), suggesting that common underlying mechanisms may support inflammatory crosstalk. This supports the concept of therapeutic approaches that target inflammasomes and their effectors and downstream targets, using precision approaches in individuals with clonal hematopoiesis.

## Methods

### Sex as a biological variable.

We focused exclusively on female mice because they are more prone to developing advanced atherosclerotic lesions. This characteristic made them particularly suitable for evaluating the experimental outcomes (necrotic core and fibrosis cap) in our studies. Prior studies have shown similar features of plaque instability in male and female *Jak2^VF^* CH mice (62).

### Mice.

WT C57BL/6J (strain no. 000664), GFP (C57BL/6-Tg(CAG-EGFP)131Osb/LeySopJ (strain no. 006567)), *Ldlr^–/–^* (strain no. 002207), and *Il1r1^–/–^* (strain no. 003245) mice were purchased from The Jackson Laboratory. *Jak2^V617F^* (*Jak2^VF^*) mice were generated and described previously (88). *Mertk*^CR^ mice were originally generated by Bishuang Cai (Icahn School of Medicine at Mount Sinai, New York, New York, USA) and Ira Tabas (Columbia University, New York, New York, USA) (52). *Ldlr^–/–^* recipient mice were fed a WD (Harlan Teklad, TD88137) for the indicated period. In the cohort treated with the TREM2-activating antibody (4D9), isotype control IgG (DC1887) and 4D9 (DC1847) mouse antibodies were injected i.p. into the mice at 10 mg/kg per week for 14 weeks. All mice were housed under a 12-hour light/12-hour dark cycle and pathogen-free conditions.

### BMT and antibody administration.

Before transplantation, 8- to 10-week-old Mx1-Cre *Jak2^VF^* and littermate control Mx1-Cre mice were i.p. injected with polyinosinic polycytidilic acid (pIpC) (250 μg/mouse) every other day for a total of 3 injections. Two weeks after administration of the last dose of pIpC, whole BM cells were isolated from the femur, hip, and tibia by centrifugation at 8,000*g* for 1 minute. Female recipient *Ldlr^–/–^* mice, aged 8–12 weeks, were lethally irradiated once with 10.5 Gy from a caesium gamma source. Within 24 hours of irradiation, BM was collected from the 6- to 10-week-old donor mice of the indicated genotypes, and the total number of BM cells was quantified. Irradiated mice were randomized to treatment groups and then received 6 million BM cells each by i.v. injection.

### BMDMs.

BM cells from 8- to 16-week-old mice were cultured in DMEM supplemented with 10% heat-inactivated FBS, 100 mg/mL streptomycin, 10 U/mL penicillin, and 20% L-929 fibroblast culture media for 5 days to induce macrophage differentiation as previously described (89).

### Atherosclerosis lesion analysis and metabolic profiling.

The aortic root from the heart was embedded in paraffin and then serially sectioned. Six sections per mouse were stained with H&E for total lesion and necrotic core area quantification. Smooth muscle fiber and collagen content staining was done using Masson’s Trichrome Staining kit (MilliporeSigma, HT15) following the manufacturer’s instructions. Total plasma cholesterol and triglyceride levels were measured using kits from Wako Diagnostics.

### Complete blood count.

Complete blood counts were performed using whole blood collected from facial vein bleeding in an EDTA-coated tube and then analyzed with the FORCYTE Veterinary Hematology Analyzer (Oxford Science).

### Immunofluorescence staining and fluorescence intensity quantification.

Paraffin-embedded slides were deparaffinized and rehydrated in Trilogy (Cell MARQUE, 920P-09). The antibodies used for staining included the following: anti–Mac-2 (Cedarlane, CL8942AP, 1:10,000); GFP (Abcam, ab13970, 1:500); MERTK (R&D Systems, BAF591, 1:200); anti–IL-1β (Abcam, ab9722 1:200); anti-TREM2 (Denali, 4D9 DC1847 or Proteintech 27599-1-AP, 1:100); anti-H3cit (Abcam, ab5103, 1:100); anti–cleaved gasdermin D (Cell Signaling Technology, 10137S, 1:100); anti-GSDMD (Santa Cruz Biotechnology, sc-393581AF647); anti-MPO (R&D Systems, BAF3667, 1:30); anti-decorin (Abcam, ab277636, 1:100); anti–α-SMA (MilliporeSigma, C6198 1:300); anti-PDGFB (Abcam, ab23914, 1:100); anti-PDGFRα (R&D Systems, AF1062, 1:100); and anti–TGF-β1 (Abcam, ab215715, 1:100). The sections were incubated with primary antibodies overnight at 4°C and then incubated with secondary antibodies for 30 minutes. Sections were mounted using DAPI and imaged using a Leica DMI6000B microscope. For TUNEL staining, a Roche In Situ Cell Death Detection Kit (MilliporeSigma) was used according to the manufacturer’s instructions. In all immunofluorescence staining, an isotype-matched normal IgG was used as the negative control. The fluorescence intensity of proteins of interest was quantified using ImageJ (NIH) according to the following steps: (a) open the immunofluorescence image in ImageJ; (b) convert the image to grayscale by selecting Image > Type > 8-bit; (c) adjust the threshold to isolate the stained areas from the background and isotype control staining by navigating to Image > Adjust > Threshold and using the slider to set the appropriate threshold levels; (d) apply the threshold to generate a binary image where the staining is highlighted; and (e) select the region of interest using the selection tools and then use Analyze > Measure to obtain the integrated intensity. MFI was calculated by dividing the integrated density (IntDen) by the area of the lesion (the Mac2^+^ macrophage in the aortic root cross sections) (mean = IntDen/area).

### Flow cytometry.

Flow cytometry was used to characterize PBMC profiles and BMDMs. For the PBMC profiles and BMDMs, lysed WBCs or BMDMs were labeled with APC/Cy7 LIVE/DEAD (Invitrogen, Thermo Fisher Scientific, MP34955) and anti-CD45 (BioLegend, 103128, 1:200), anti-CD11b (BioLegend, 101216, 1:200), anti–Gr-1 (BioLegend, 108445, 1:200), anti-CD115 (BioLegend,134410, 1:100), anti-TREM2 (R&D Systems, FAB17291A, 1:100), and anti-MERTK (Thermo Fisher Scientific, 12-5751-82, 1:100). Flow cytometry was performed using the LSRCanto or LSR II (BD), and data were analyzed with FlowJo software (BD). Isotype-matched normal IgG was used as the control in each flow cytometry assay.

### Immunoblotting.

BMDMs were lysed in RIPA buffer containing protease phosphatase inhibitors on ice for 10 minutes and then centrifuged at 14,000*g* for 5 minutes to generate protein lysates. Nuclear protein lysates were extracted using a kit from Abcam (ab113474). The protein concentration was determined by BCA assays and then mixed with 4X Laemmli sample buffer and heated at 95°C for 5 minutes. Protein was separated by 4%–20% gradient SDS-PAGE and transferred onto nitrocellulose membranes. Then, the membranes were blocked with 5% nonfat milk in TBST and immunostained with the primary antibodies anti–p-Syk (Cell Signaling Technology, 2710s, 1:1,000), anti-Syk (Cell Signaling Technology, 13198, 1:1,000), anti–p-AKT (Cell Signaling Technology, 4060s, 1:1,000), anti-AKT (Cell Signaling Technology, 4685, 1:1,000), anti–cleaved gasdermin D (Cell Signaling Technology, 10137S, 1:1,000), anti–p–β-catenin (Cell Signaling Technology, 5651, 1:1,000), anti–β-catenin (Cell Signaling Technology, 8480S, 1:1,000), anti-PDGFB (Abcam, ab23914,1:1,000), anti–histone H3 (Cell Signaling Technology, 4499, 1:1,000), anti-MERTK (R&D Systems, AF591, 1:1,000), and β-actin (Cell Signaling Technology, 4970s, 1:5,000) at 4°C overnight and detected using HRP-conjugated secondary antibodies.

### Human atherosclerotic cells analysis.

Bulk and scRNA-Seq was performed as described previously (90). Briefly, total RNA from tissue biopsies obtained from patients undergoing carotid endarterectomy was used to generate libraries that were sequenced on the Illumina NovaSeq platform (NovaSeq6000, Illumina), using the TruSeq stranded total RNA kit and TruSeq stranded mRNA kit (Illumina). Raw data (raw reads) in the FASTQ format were aligned, and quality control (QC) and differential gene expression (DEG) analyses were performed by FiosGenomics (Edinburgh). To correlate the gene expression of PDGFB and TREM2, Pearson’s correlation analysis was performed on 202 patients’ samples. The pipeline was implemented using Python 3.9 and the Scipy 1.12.0 library.

scRNA-Seq. Human atherosclerotic tissue was minced and digested using the Multi Tissue Dissociation Kit 2 (Miltenyi Biotec, 130-110-203), the GentleMACS Dissociator (Miltenyi Biotec, 130-093-235), GentleMACS C tubes (Miltenyi Biotec, 130-096-334), and the 37 C_Multi_G program, all according to the manufacturer’s instructions. The cell suspension was strained (70 μm, 40 μm), and a Dead Cell Removal kit (Miltenyi Biotec, 130-090-101) with MS Columns (Miltenyi Biotec,130-042-201) were used. Cells were resuspended in PBS plus 0.04% BSA.

Single-cell capture and library preparation were performed according to the manufacturer’s instructions (10X Genomics, CG000204 Rev D). Libraries from individual samples were multiplexed into 1 lane before sequencing on an Illumina NovaSeq 6000 instrument. Data were aligned and preprocessed and deposited in the Gene Expression Omnibus (GEO) database (GEO GSE247238).

Data preprocessing of scRNA-Seq data on 18 patients’ samples (all but CAR1_Seq1) was conducted using Python 3.9 and the ScanPy 1.9.6 library, in which mitochondrial and ribosomal RNAs were filtered out, focusing exclusively on highly variable genes. For clustering cell populations, the Leiden algorithm was applied.

### scRNA-Seq analysis.

FASTQ files of the multiplexed scRNA-Seq data for the aortic cells were processed with Cell Ranger, version 6.1.2. The mm10-2020-A reference transcriptome (GENCODE vM23/Ensembl98) was used to align the sequencing reads, and cells were assigned to their respective samples of origin using 4 unique hashtag oligonucleotides (HTOs). Seurat 4.3.0 (91) in R software, version 4.1.0, was then utilized to analyze the unique molecular identifier (UMI) count matrix for each sample. Cells expressing fewer than 200 genes and containing more than 10% mitochondrial genes, as well as genes expressed in fewer than 10 cells, were excluded. For each sample, additional preprocessing steps were carried out according to sequencing depth, with the criteria detailed below. In each dataset, gene counts were normalized to the total number of genes per cell and were then scaled by a factor of 10,000 and subjected to a natural log transformation. The top 1,000 variable genes were selected from each single-cell sample. The 4 datasets were then integrated separately for aortic cells, utilizing the top 2,000 highly variable genes identified across the datasets and 20 dimensions of integration anchors. In the examination of aortic scRNA-Seq data, the first 15 principal components (PCs) and the 20 nearest neighbors were used to develop the shared nearest neighbor (SNN) graph. A resolution setting of 0.5 was then used through Louvain clustering to categorize the various cell types. Differential gene expression (DEG) analysis was performed through the Wilcoxon rank-sum test to identify the most significantly expressed genes by contrasting 1 cluster against all others. Genes that were expressed in over 25% of cells, exhibited a minimum fold change of 1.5, and achieved a Bonferroni corrected *P* value of less than 0.05 were classified as differentially expressed for each cell type.

### Ligand-receptor interaction analysis of scRNA-Seq data.

A ligand-receptor interaction analysis was performed using CellPhoneDB, version 5.0.0 (92), in Python, version 3.8. The mouse gene symbols were converted to human gene symbols using biomaRt, version 2.48.3 (93), in R, excluding any mouse genes lacking their corresponding human orthologs. Ligand-receptor interactions were detected through the analysis of ligand and receptor expression in macrophages, SMCs, and fibroblasts. Macrophages were compared with SMCs or fibroblasts to identify potential interactions with the TGF-β family (*Tgfb1*, *Tgfb2*, *Tgfb3*) or PDGF subunit B (*Pdgfb*). The mean expression levels of ligands and receptors in the interacting populations were determined by permuting the population labels of all cells 1,000 times. Ligands and receptors were considered in the analysis if they were expressed in more than 10% of the cells of the corresponding cell type. Dot plots of ligand-receptor interactions were generated using ktplots, version 2.1.0 (92), in R.

### Preprocessing parameters for mouse aortic cell scRNA-Seq data.

Genes expressed in fewer than 10 cells and cells expressing fewer than 200 genes were excluded for data QC purposes. In the LAB *Jak2^VF^* plus IgG aortic cells sample, the maximum number of UMIs was 15,000, and the maximum number of genes was 2,500. This sample contains 13,373 genes and 2,898 cells. In the *Jak2^VF^* plus 4D9 sample, the maximum number of UMIs was also 15,000, and the maximum number of genes was 2,500. This sample contained 13,171 genes and 3,075 cells. Ten percent of the reads were mitochondrial in both samples.

### Real-time qPCR.

After treatment, the cells were washed with PBS. RNA was extracted from samples using the RNeasy Mini kit (QIAGEN, 74106). The RNA concentration was assessed with the Thermo Scientific NanoDrop spectrophotometer. cDNA was synthesized, qPCR was performed using the Real-Time PCR system (Applied Biosystem) and SYBR Green Master Mix. Primers were purchased from Integrated DNA Technologies. The primer sequences for qPCR were as follows: *Tgfb1*, forward, CTCCCGTGGCTTCTAGTGC, reverse, GCCTTAGTTTGGACAGGATCTG; *Tgfb2*, forward, CTTCGACGTGACAGACGCT, reverse, GCAGGGGCAGTGTAAACTTATT; *Tgfb3*, forward, CCTGGCCCTGCTGAACTTG, reverse TTGATGTGGCCG-AAGTCCAAC; *Fgf12*, forward, CTCGGGGTGTTCAGCAAAGT, reverse, TTTCGTCCTTGGTCCCATCAA; *PAI-1*, TTCAGCCCTTGCTTGCCTC, reverse, ACACTTTTACTCCGAAGTCGGT; *Pdgfb*, forward, CATCCGCTCCTTTGATGATCTT, reverse, GTGCTCGGGTCATGTTCAAGT; *Ltbp1*, forward, CGAGCATCTGTAAAGTGACCTG, reverse, CGTGCTGGTAAAGTTTGGGC; *Mmp7*, forward, CTGCCACTGTCCCAGGAAG, reverse, GGGAGAGTTTTCCAGTCATGG; *Ltbp2*, forward, AACAGCACCAACCACTGTATC, reverse, CCTGGCATTCTGAGGGTCAAA; *Mmp9*, forward, CTGGACAGCCAGACACTAAAG, reverse, CTCGCGGCAAGTCTTCAGAG; *Ltbp3*, forward, CTTGCCCCACAAGGAGAGTC, reverse, CACTTCTGCCGAGATTTGTCC; *Timp1*, forward, GCAACTCGGACCTGGTCATAA, reverse, CGGCCCGTGATGAGAAACT; *Ltbp4*, forward, CTGCGTCTCCAACGAGAGC, reverse, TCTGGGGGCAGTGTAGAGC; *Timp2*, forward, TCAGAGCCAAAGCAGTGAGC, reverse, GCCGTGTAGATAAACTCGATGTC.

### Statistics.

Data that passed the normality test were analyzed using a 2-tailed Student’s *t* test for 2 groups; 1-way ANOVA with Tukey’s post hoc analysis for more than 2 groups; or 2-way ANOVA with Šidák’s post hoc analysis for 2 factors. Data that were not normally distributed were analyzed using the nonparametric Mann-Whitney *U* test, or, for more than 2 groups, by Kruskal-Wallis test with post hoc analysis using the Dunn test. Outliers were removed using Grubb’s test (Prism). A *P* value of less than 0.05 was considered significant. Statistical analyses were conducted and analyzed using GraphPad Prism (GraphPad Software).

### Study approval.

Human carotid artery samples were collected within the Munich Vascular Biobank, upon patients’ informed consent, as described previously (94). Use of the Biobank data was approved by the local hospital ethics committee (2799/10, Ethics Committee of the Faculty for Medicine at the Technical University of Munich, Munich, Germany) and was in accordance with the Declaration of Helsinki (90). All protocols were approved by the IACUC of Columbia University.

### Data availability.

Values for all data points associated with the manuscript and supplemental results are provided in the Supporting Data Values file. The scRNA-Seq data from mice have been deposited in the NCBI’s Gene Expression Omnibus (GEO) database (GEO GSE280703). Additional information and detailed methods are available upon request to the corresponding authors.

## Author contributions

WL designed and performed experiments, analyzed data, and wrote the manuscript. BH designed and performed the experiments and edited the manuscript. EK designed and conducted the scRNA-Seq analysis. JP, JLW, and LM conducted studies of human carotid arteries and analysis. MY, CCH, and TX performed the experiments and edited the manuscript. KS and CH generated and provided IgG and 4D9 antibodies, designed the experiments, and edited the manuscript. MPR, IT, and NW shared the reagents, designed experiments, and provided scientific feedback about the manuscript. ART designed experiments, wrote the manuscript, and supervised and directed the project.

## Supplementary Material

Supplemental data

Unedited blot and gel images

Supporting data values

## Version 1 11/12/2024

In-Press Preview

## Version 2 01/02/2025

Electronic publication
