# Supplementary material for: Inflammatory crosstalk impairs phagocytic receptors and aggravates atherosclerosis in clonal hematopoiesis in mice
Source: J Clin Invest. 2024 Nov 12;135(1):e182939. doi: 10.1172/JCI182939 (PMC11684819; doi:10.1172/JCI182939)

Supplemental Figure 1 (Related to Figure 1)

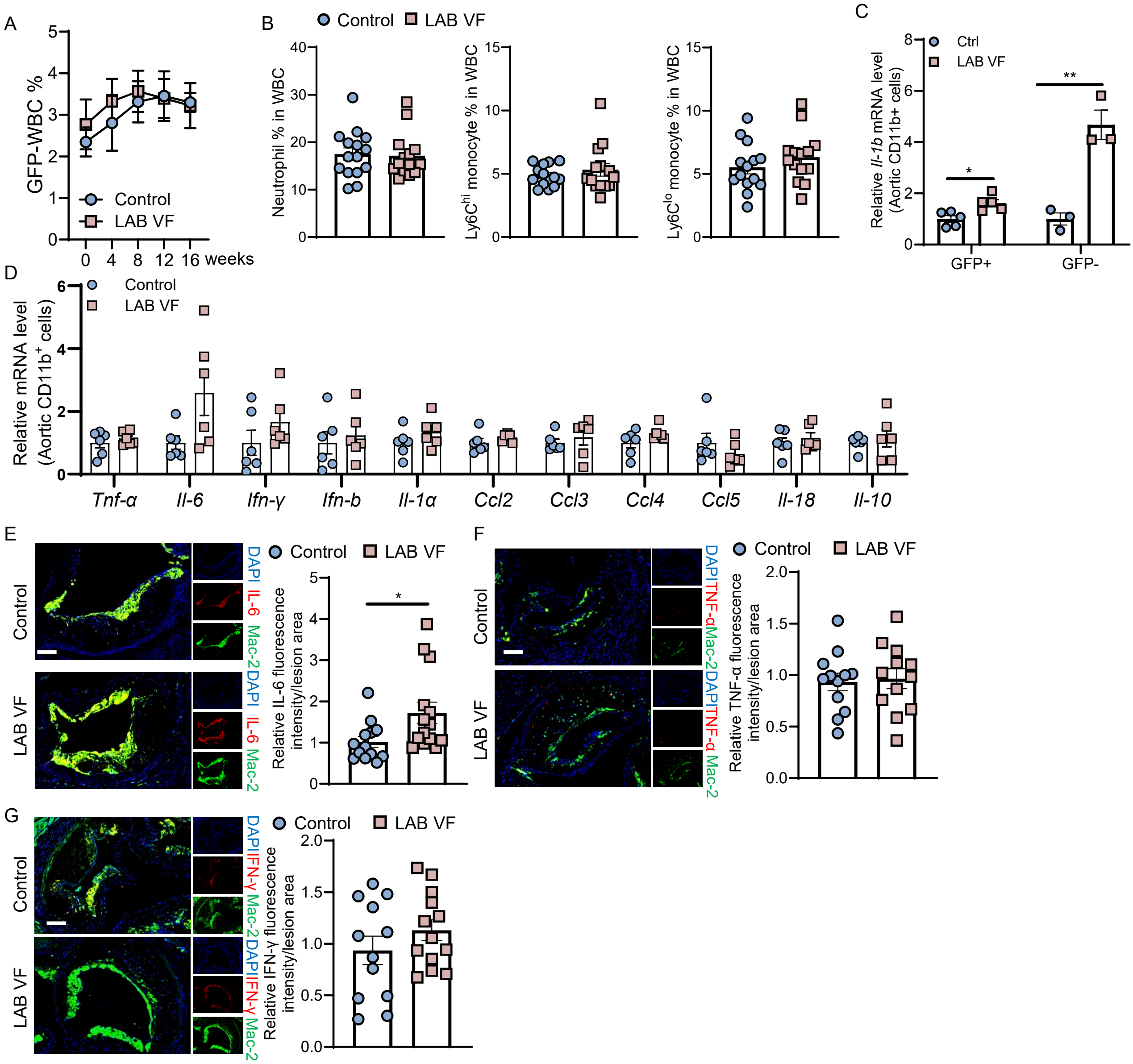

Supplemental Figure 2 (Related to Figure 2-3)

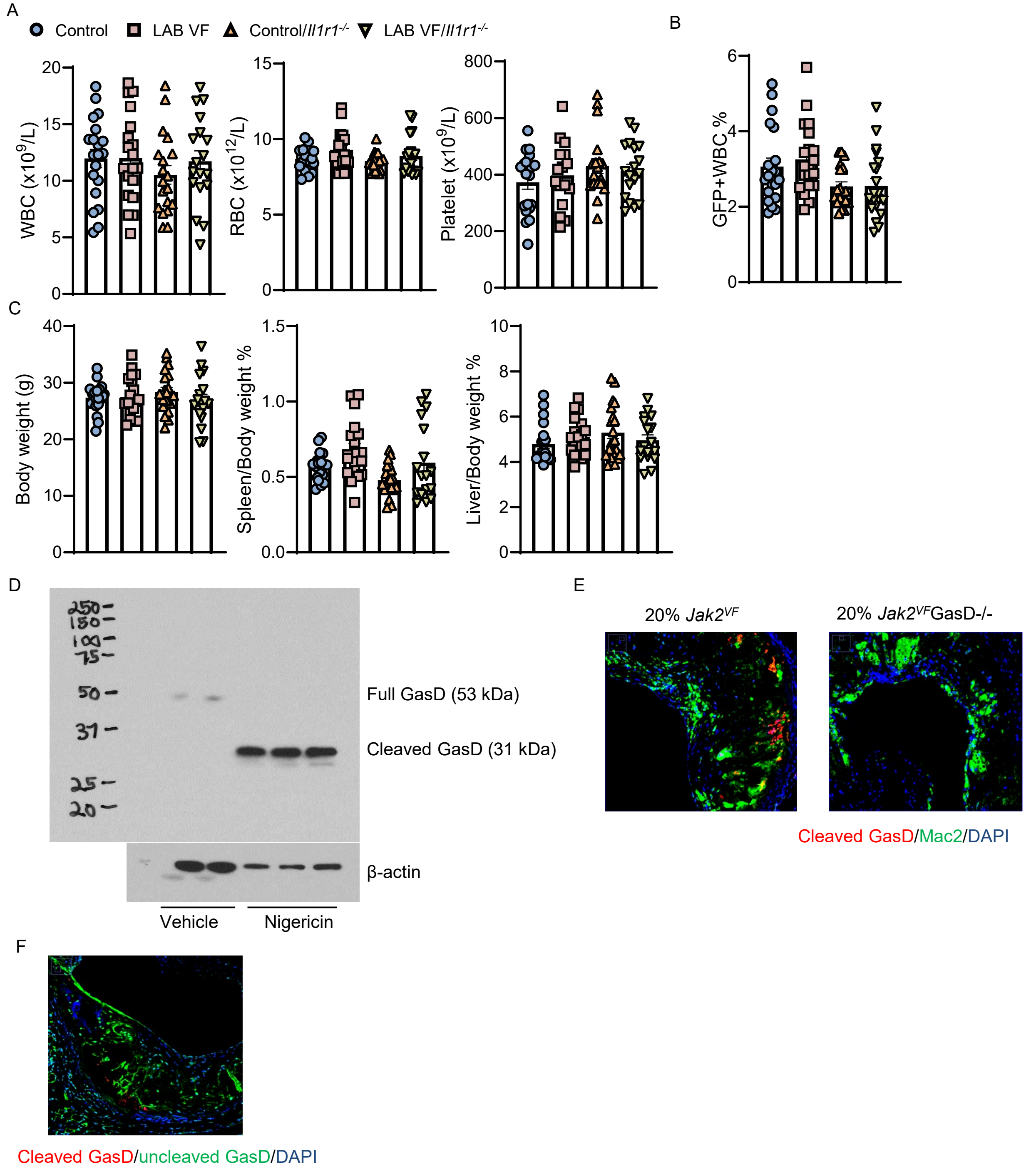

Supplemental Figure 3 (Related to Figure 4-5)

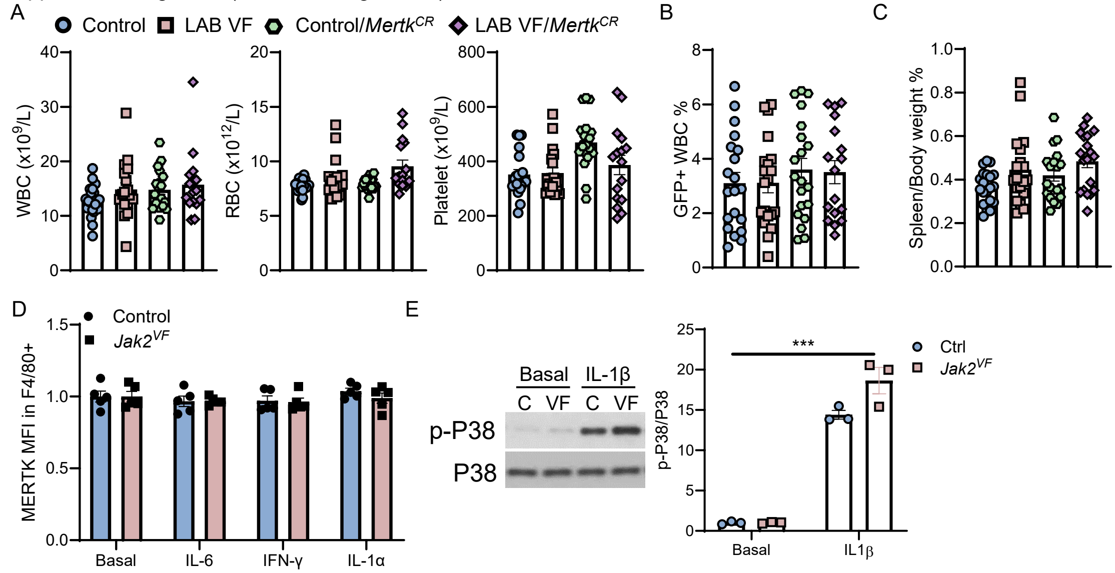

Supplemental Figure 4 (Related to Figure 6-7)

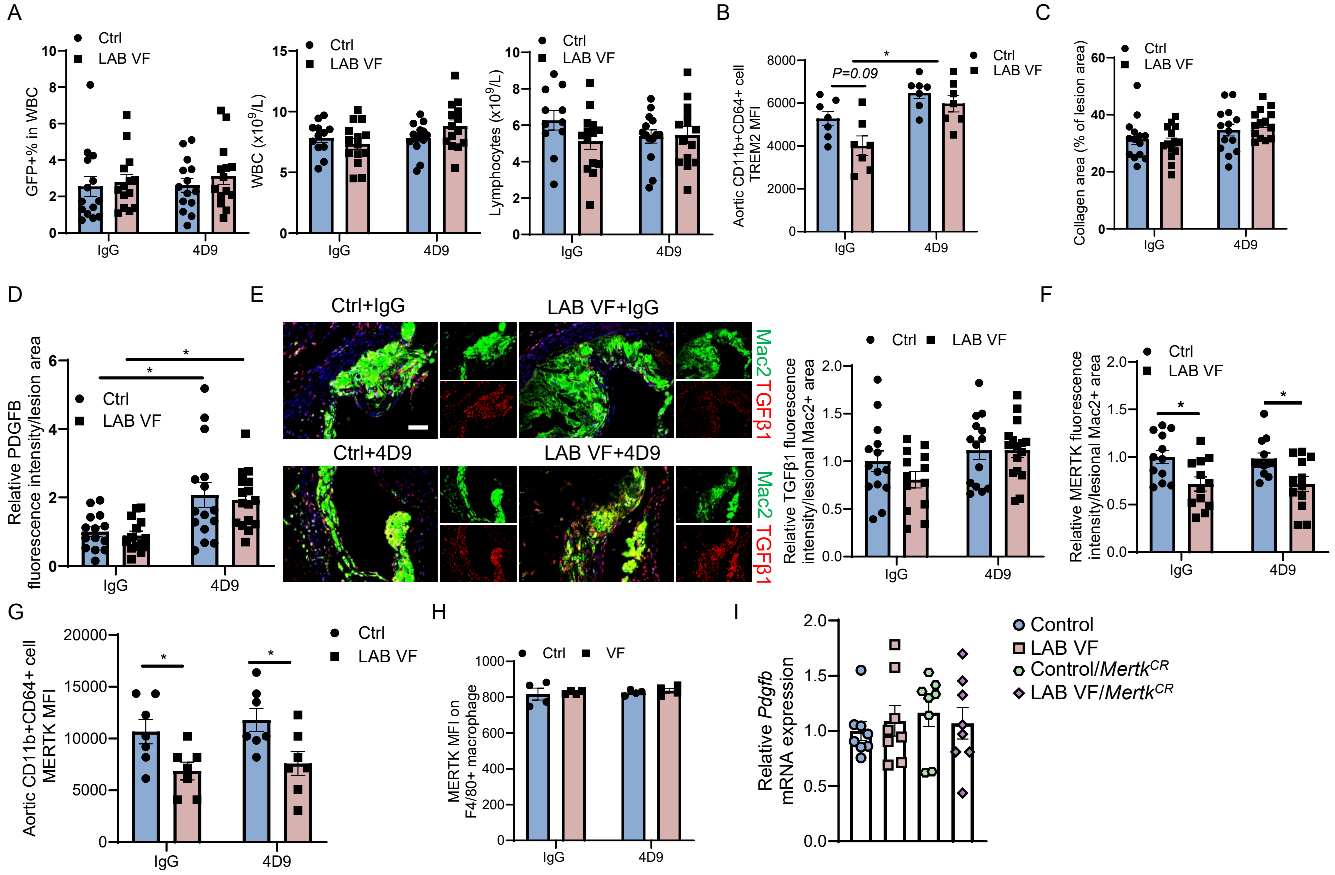

Supplemental Figure 5 (Related to Figure 7)

A

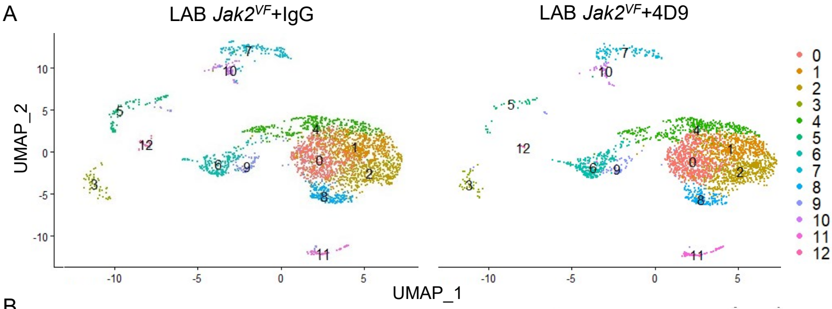

Cl	Cell Type
0	SMC 1
1	SMC 2
2	SMC 3
3	B
4	SEM
5	T 1
6	Fibroblast 1
7	Macrophage
8	Pericytes?
9	Fibroblast 2
10	Monocyte
11	Endothelial
12	T 2 (Proliferative)

B

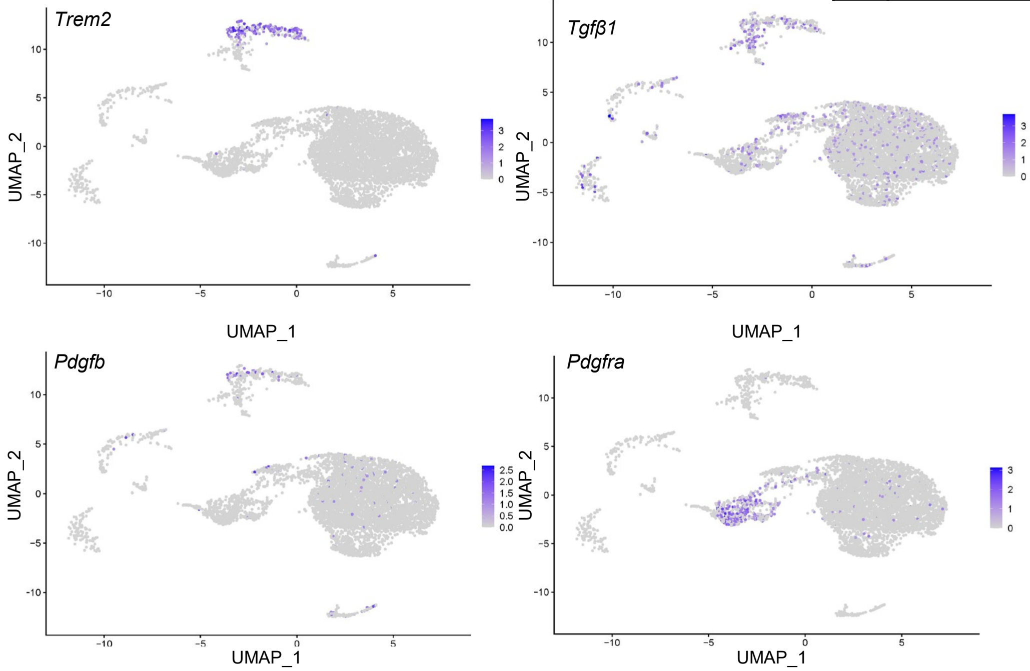

Supplemental Figure 6

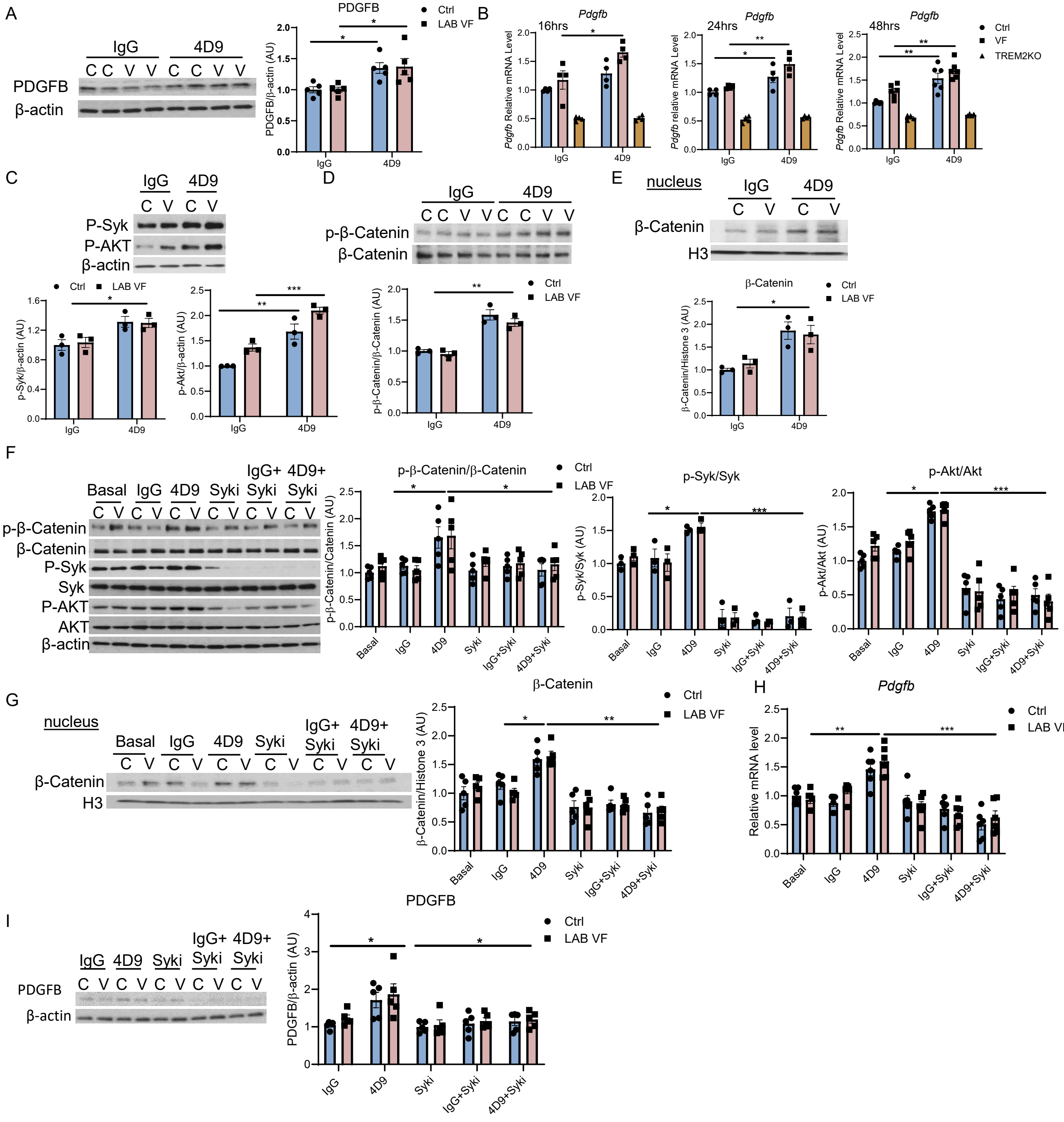

Supplement: Supplemental data [file jci-135-182939-s022.pdf]
