# Supplementary material for: Inflammatory crosstalk impairs phagocytic receptors and aggravates atherosclerosis in clonal hematopoiesis in mice
Source: J Clin Invest. 2024 Nov 12;135(1):e182939. doi: 10.1172/JCI182939 (PMC11684819; doi:10.1172/JCI182939)

Full unedited blot for Figure 5

B

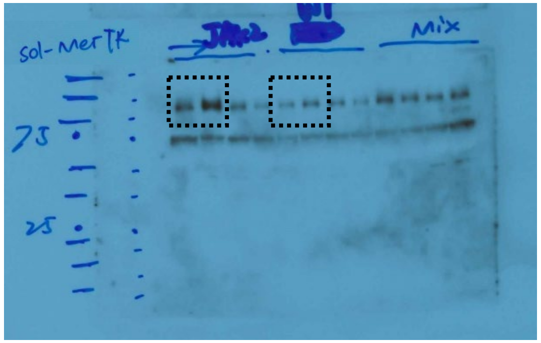

C

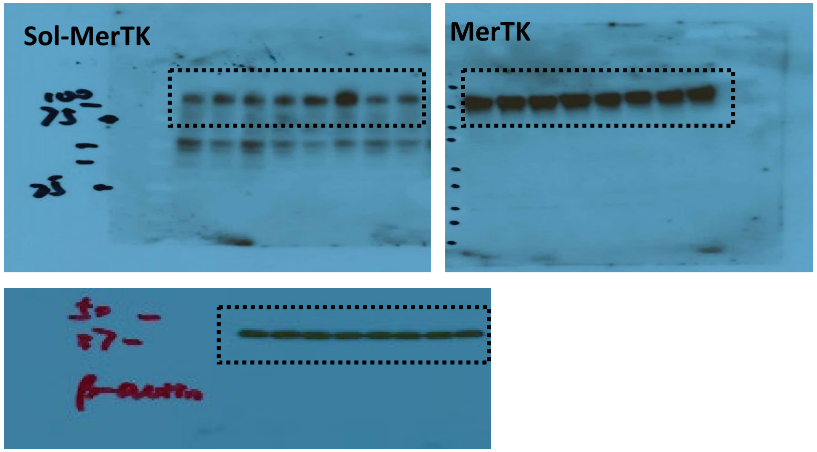

Full unedited blot for supplemental Figure 2D

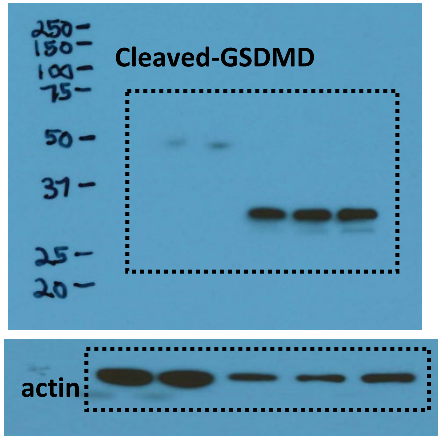

Full unedited blot for supplemental Figure 3E

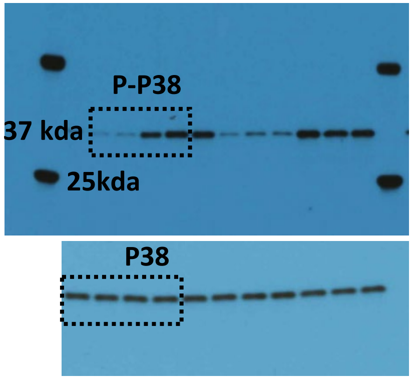

Full unedited blot for supplemental Figure 6

A

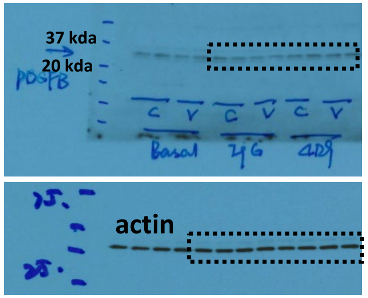

C

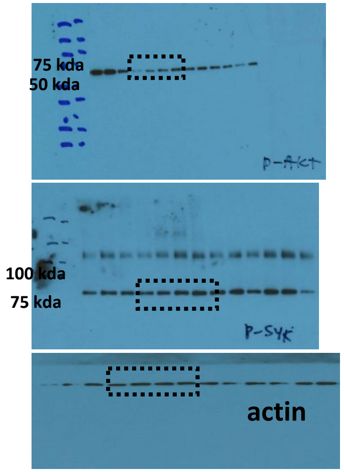

D

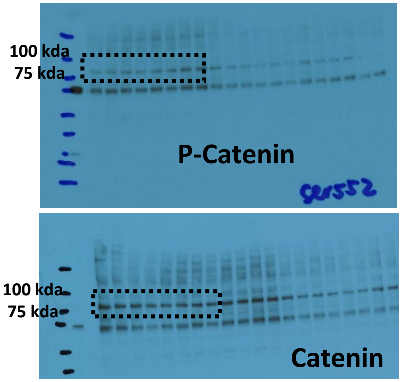

F

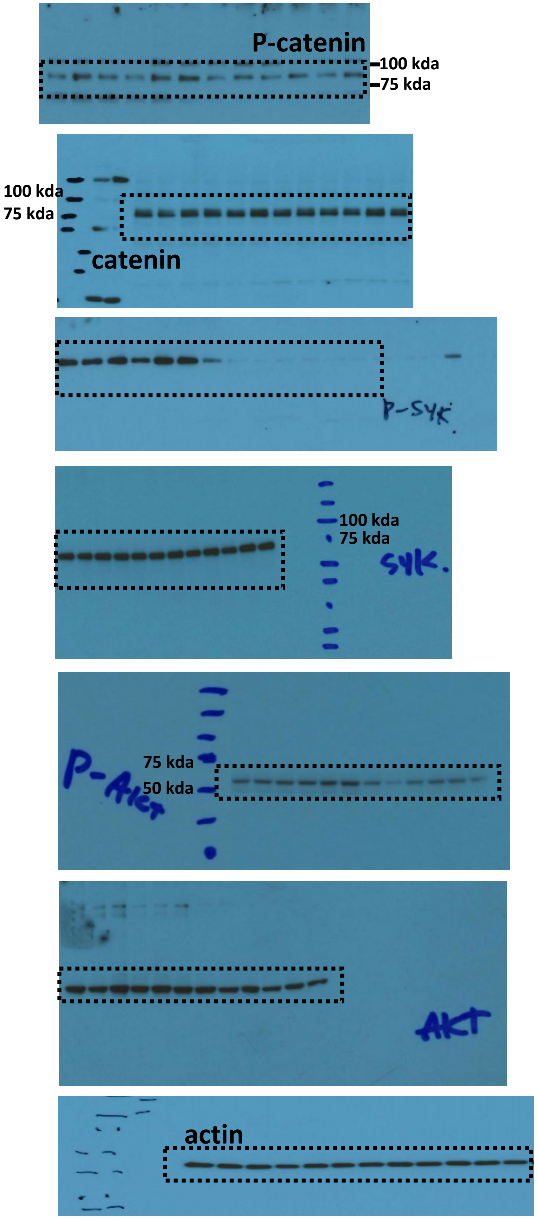

E

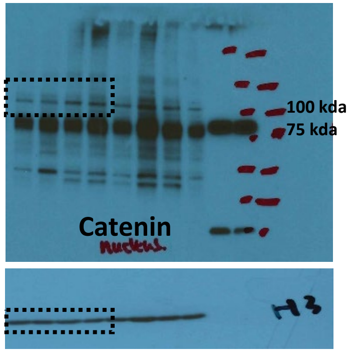

G

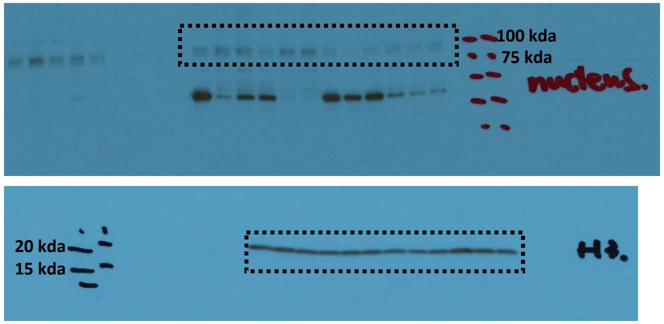

I

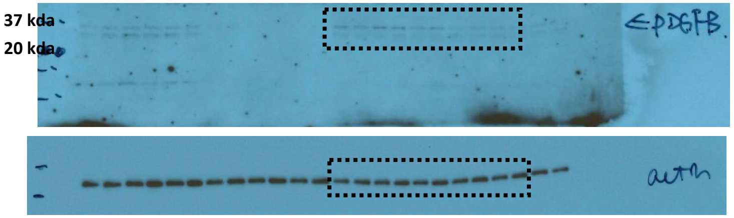

Supplement: Unedited blot and gel images [file jci-135-182939-s023.pdf]
